# EZH2 PROTACs outperform catalytic inhibitors in prostate cancer by targeting a methylation-independent function of PRC2

**DOI:** 10.1038/s41388-025-03662-z

**Published:** 2026-01-07

**Authors:** Wanqing Xie, Qi Chu, Lourdes Brea, Guihua Zeng, Yuan Wang, Xiaodong Lu, Mohan Zheng, Corinne R. Ley, Zhiquan Lei, Hongshun Shi, Joshua L. Zhu, Lihu Gong, M. Cynthia Martin, Xianglin Shi, Galina Gritsina, Arabela A. Grigorescu, Hana Chandonnet, Xin Liu, Jonathan C. Zhao, Gary E. Schiltz, Jindan Yu

**Affiliations:** 1 Department of Urology, Emory University School of Medicine, Atlanta, GA USA; 2 Division of Hematology/Oncology, Department of Medicine, Northwestern University Feinberg School of Medicine, Chicago, IL USA; 3 Department of Chemistry, Northwestern University, Evanston, IL USA; 4 Cecil H. and Ida Green Center for Reproductive Biology Sciences, University of Texas Southwestern Medical Center, Dallas, TX 75390 USA; 5 Department of Molecular Biosciences, Northwestern University, Evanston, IL USA; 6 Department of Human Genetics, Emory University School of Medicine, Atlanta, GA USA; 7 Winship Cancer Institute, Emory University School of Medicine, Atlanta, GA USA; 8 Department of Pharmacology, Northwestern University, Chicago, IL USA; 9 Robert H. Lurie Comprehensive Cancer Center, Northwestern University, Chicago, IL USA

**Keywords:** Prostate cancer, Drug regulation

## Abstract

Enhancer of Zeste Homolog 2 (EZH2) is the enzymatic subunit of the Polycomb Repressive Complex 2 (PRC2). It catalyzes H3K27 methylation for epigenetic silencing of tumor suppressors and critically drives prostate cancer (PCa) progression. However, inhibitors of EZH2 catalytic function (EZH2i), such as EPZ-6438, showed limited efficacy in PCa. Here, we designed and developed a series of VHL-based proteolysis-targeting chimera (PROTAC) degraders of EZH2 using EPZ-6438 as a ligand and identified PROTAC-6272 as a lead compound. PROTAC-6272 effectively degraded EZH2 and other PRC2 subunits across diverse PCa cell lines. However, PROTAC-6272 and other similar EZH2i-based PROTACs were consistently unable to decrease androgen receptor (*AR*), a gene that is directly activated by solo EZH2. Mechanistically, EZH2 PROTACs failed to degrade EZH2 coactivators, such as p300, due to their inability to engage EZH2 outside of the PRC2 complex. Nevertheless, PROTAC-6272 exhibited anti-proliferative activities superior to EPZ-6438 in some PCa models, wherein it induced p21 expression and cellular senescence by disrupting a methylation-independent PRC2 function. In summary, while EZH2i-based PROTACs failed to target the PRC2-independent functions of EZH2, they confer added benefits over EPZ-6438 by abolishing a polycomb-dependent but methylation-independent function of EZH2, offering therapeutic advantages in some PCa.

## Introduction

Prostate cancer (PCa) is the second leading cause of cancer-related deaths among men, with approximately 350,000 deaths reported globally each year [1]. Although surgery, radiation, and androgen deprivation therapy (ADT) have shown success in extending PCa patient lifespans, resistance often occurs, leading to metastatic castration-resistant prostate cancer (CRPC), which is accountable for a majority of PCa mortality [2]. Enhancer of Zeste Homolog 2 (EZH2) is the catalytic subunit of the polycomb repressive complex 2 (PRC2), which methylates lysine 27 of histone H3 to promote epigenetic silencing [3]. Aberrant EZH2 expression and enhanced PRC2 catalytic activity are frequently observed in metastatic CRPC and play critical roles in driving PCa progression [4, 5]. As a result, there has been a huge endeavor in developing EZH2 inhibitors for the treatment of advanced PCa, as well as other aggressive cancers exhibiting aberrant EZH2 activity. Given EZH2’s essential role in mediating H3K27 trimethylation and the significance of this histone mark in regulating cellular differentiation, proliferation, and tumorigenesis, numerous inhibitors specifically targeting EZH2’s enzymatic function have been developed [6–8]. EPZ-6438 is a selective inhibitor of EZH2 histone methyltransferase activity, which binds to the SET domain of EZH2 to compete with S-adenosylmethionine (SAM). EPZ-6438 has shown great promise in preclinical studies and clinical trials for its ability to inhibit PRC2 activity, resulting in reduced tumor growth and arrested progression in certain cancer types [8, 9]. In 2020, the first-in-class EZH2 inhibitor EPZ-6438 (tazemetostat) was approved by the FDA for the treatment of metastatic and locally advanced epithelioid sarcoma [10].

However, growing evidence suggests that EZH2’s oncogenic role extends beyond its enzymatic activity, involving Polycomb- and methylation-independent mechanisms [11–14]. Of particular relevance to PCa, EZH2 has been shown to directly induce the expression of androgen receptor (AR) by functioning as a transcriptional activator. Importantly, enzymatic EZH2 inhibitors are unable to block this PRC2-independent role of EZH2 as an activator [14] and thus have shown very limited efficacy in PCa [15, 16]. These findings call for compounds that could block both the PRC2-dependent and -independent roles of EZH2. To this end, several EZH2-targeting Proteolysis-Targeting Chimeras (PROTACs) have recently been developed with the goal of degrading all EZH2 proteins. These reported EZH2 PROTACs include compounds with EPZ-6438 attached to ligands for CRBN [17, 18] and VHL [19, 20], along with the potent EZH2 inhibitor GSK126 attached to a ligand for CRBN [21]. In addition to the commonly employed CRBN and VHL E3 ligases, others have reported on the use of MDM2 as the E3 ligase to facilitate degradation of EZH2 [22], along with hydrophobic tag degraders of EZH2 using an adamantyl group [23]. Of note, most of the studies have reported the efficacy of PROTACs in suppressing lymphoma and/or breast cancer [17–22, 24, 25]. Only one PROTAC, MS177, which was shown to degrade p300, c-Myc, and AR, exhibited anti-proliferative effects in PCa cells [21, 26]. While compound MS177 is a PROTAC that recruits CRBN, our compound (PROTAC-6272) recruits VHL. The use of different E3 ligases in PROTACs may result in some distinct downstream effects, offering a more comprehensive and robust understanding of the multifaceted roles EZH2 plays in prostate cancer.

In this study, we developed a potent EZH2 PROTAC, PROTAC-6272, composed of EPZ-6438 and a VHL-targeting ligand. We demonstrated that PROTAC-6272 induced EZH2 degradation and drastically decreased other PRC2 subunit proteins. However, it failed to decrease AR, which is due to its inability to target EZH2 in the co-activator complex. Interestingly, PROTAC-6272 exhibited a significant inhibitory effect on the proliferation of some PCa cell lines through rapid induction of cellular senescence and up-regulation of p21 upon EZH2-PRC2 degradation. These data suggest that PROTACs derived from EZH2 enzymatic inhibitors, albeit unable to degrade non-PRC2 EZH2, have therapeutic benefits in PCa that are sensitive to the induction of cellular senescence.

## Materials and methods

### Compound synthesis

The syntheses of compounds 6272 and their analytical data are described in the Supplementary Information.

### Cell lines, chemical reagents, and antibodies

PCa cell lines LNCaP, C4-2B, 22Rv1, DU145, PC-3, LNCaP-abl, VCaP, and the human normal prostate epithelial cell line RWPE-1 were obtained from the American Type Culture Collection (ATCC). LNCaP-95 was provided by Dr. Jun Luo. LNCaP, C4-2B, 22Rv1, DU145, and PC-3 were maintained in RPMI1640 or Dulbecco’s Modified Eagle’s Medium (DMEM) with 10% FBS and 1% penicillin-streptomycin. RWPE-1 cells were maintained in keratinocyte serum-free medium with 0.05 mg/mL BPE and 5 ng/mL epidermal growth factor. LNCaP-abl cells were cultured in phenol red–free RPMI 1640 medium supplemented with 10% charcoal-stripped FBS and 1% penicillin-streptomycin. All cells were authenticated within 6 months of growth, and cultures were frequently tested for potential mycoplasma contamination. The following antibodies were used: rabbit anti-EZH2 (5246S; CST, MA, USA); rabbit anti-SUZ12 (3737S; CST); sheep anti-EED (AF5827; R&D, MN, USA); rabbit anti-VHL (68547S; CST); rabbit anti-AR (06-680; Millipore, MA, USA); rabbit anti-H3K27me3 (9733S; CST); rabbit anti-Histone3 (ab1791; Abcam, UK); rabbit anti-p300 (54062S; CST); rabbit anti-c-Myc (ab32072; Abcam); rabbit anti-p21 (2947S; CST); rabbit anti-c-Myc (ab32072; Abcam); rabbit anti-HA (ab9110; Abcam); mouse anti-α-tubulin (sc-32293; Santa Cruz Biotechnology, TX, USA); rabbit anti-GAPDH (2118; CST) and rabbit anti-Ki67 (ab16667; Abcam) for Western blotting and IHC experiments.

### diaPASEF quantitative LCMS proteomics and data analysis

The data and method are described in the Supplementary Information.

### Protein purification and Isothermal Titration Calorimetry (ITC)

The FLAG-tagged human EZH2 was expressed in Sf9 insect cells. The insect cells were harvested 48 h post-infection, following sonication in the lysis buffer. The clarified supernatant was loaded onto anti-FLAG beads (ThermoFisher Scientific, Cat No. A36797). The captured protein was eluted; a broad elution profile was observed. The EZH2 (solo EZH2) and ternary human PRC2 complex, EZH2–EED–SUZ12 (VEFS), containing a full-length EZH2 (residues 1–746) fused to the VEFS domain of SUZ12 (residues 543–695) and a full-length EED (residues 1–441), was expressed in the S. cerevisiae CB010 strain as previously reported [27]. Fractions containing the purified protein complex were pooled, concentrated, and stored at –80 °C.

The ITC data for the interaction of the EPZ-6438 compound with the EZH2 protein and PCR2-EZH2 complex were processed and analyzed with the MicroCal PEAQ-ITC (Malvern Instruments Ltd.). 40 μL of the EPZ-6438 solution was loaded into a titrating syringe. 280 μL of the corresponding protein solution (EZH2 or PRC2-EZH2 complex) was placed in the ITC cell. After the instrument was equilibrated at 298 K and 900 rpm syringe rotational speed, a first injection of 0.1 μL was performed, followed by a series of 1.5 μL injections spaced at 120 sec. For each injection, the enthalpy change (ΔHi) was calculated by integrating the injection peak and normalizing by injection volume and concentration. The average residual heat was subtracted from the normalized ΔHi values.

### Cell titer glo and incucyte assay

Cell proliferation was measured with CellTiter-Glo® 2.0 (Promega, Cat No. G9242), as described by the manufacturer. Briefly, collect the cell medium and mix it with CellTiter-Glo® 2.0 reagent in a 1:1 ratio. Allow the plate to incubate at room temperature for 10 min to stabilize the luminescent signal. The luminescence was measured using the KC4 microplate reader (BioTek Instruments) and normalized to the media control. IC50 values were calculated using nonlinear regression analysis with a variable slope model (log[inhibitor] vs. normalized response) in GraphPad Prism. For the Incucyte assay, cells were seeded in 96-well plates and allowed to stabilize for 24 h. Cells were then treated with 1 µM of DMSO, PROTAC-6272, and EPZ-6438 and incubated in the Incucyte live imaging chamber (Sartorius). Images were taken every 2 h up to 9 days. Cell proliferation rate was analyzed by IncuCyte live imager software.

### Western blotting and co-immunoprecipitation

Total protein lysate cells were washed once in PBS and lysed in 1 × SDS lysis buffer (20% SDS from Amresco, 10% glycerol from Thermo Fisher Scientific, 62.5 mM TRIS-HCL pH6.8 from Bio-Rad) supplemented with protease inhibitor (Roche). For coimmunoprecipitation, C4-2B were treated with DMSO or PROTAC-6272 (0.5uM) for 3 h, and cell pellets were lysed in Co-IP buffer (50 mM tris-HCl, pH 7.4 from Life Technology, 150 mM NaCl from VWR, 1 mM EDTA from Life Technology, 1% Triton X-100 from Sigma-Aldrich) supplemented with protease inhibitor (Roche). Whole lysates were incubated with primary antibodies overnight at 4 °C with agitation, followed by 1 h incubation with protein G-conjugated magnetic beads for mouse-derived Ab and protein A-conjugated for rabbit-derived Ab (SureBeads; Bio-Rad). Bound proteins were eluted with 1.5 × sample buffer for 10 min at 95 °C with shaking at 1050 rpm. The eluted protein complex was resolved in 7–10% SDS-PAGE gel and subjected to immunoblotting.

### RNA isolation, quantitative RT-PCR, and RNA sequencing

Total RNA was isolated from cells using NucleoSpin RNA isolation kit (Takara). ReverTra Ace qPCR RT Master Mix kit (Toyobo) was used for RNA reverse transcription. Quantitative real-time PCR (qRT-PCR) was conducted with 2× Universal SYBR Green Fast qPCR Mix (Abclonal) on a StepOnePlus Real-Time PCR System (Applied Biosystems). RNA-seq reads were mapped to the NCBI human genome GRCh38 using STAR version 2.7.10. Gene raw counts were calculated by STAR. FPKM values (Fragments Per Kilobase of transcript per Million mapped reads) were calculated using an in-house Perl script. Differential gene expression was performed with the DESeq2 package (version 1.46.0) in R (Bioconductor), which applies shrinkage estimation to dispersions and fold-changes for improved stability and interpretability. Gene set enrichment analysis (GSEA) was performed using the clusterProfiler package (version 4.14.0) in R (Bioconductor), with genes ranked by log2 fold change. Enrichment scores and statistical significance were calculated to identify pathways and biological processes that were significantly affected by the treatments.

### IHC staining

IHC was performed on paraffin-embedded tissue slides. Following antigen retrieval using citrate buffer (Invitrogen), the slides were permeabilized with 0.5% Triton X-100 for 15 minutes. Subsequent steps were carried out using a ready-to-use IHC kit (Vector Laboratories) according to the manufacturer’s instructions. Counterstaining was performed with hematoxylin, followed by ethanol dehydration, xylene clearing, and mounting with Permount (Fisher Chemical). Slides were imaged using an Olympus BX41 microscope equipped with an Olympus UTV 0.5XC3 camera.

### Xenograft tumor growth

A total of 15 NSG male mice aged 8 weeks were bought from Charles River. Sample size is estimated based on previous similar studies[28]. 1 × 10^6^ VCaP cells in 50% solution of Cultrex UltiMatrix (R&D) in PBS were inoculated s.c. into mice left dorsal flank. Once the tumors reached 100 mm^3^, tumor-bearing mice were randomized into 3 groups, vehicle and PROTAC-6272 (75 mg/kg) by IP injection, and EPZ-6438 (250 mg/kg) by oral gavage for 18 days twice a day. Tumor size was measured twice a week and calculated by the formula (length (mm)×width2 (mm2) × 0.5). At the endpoint, mice were euthanized, and tumors were excised, fixed in 10% formalin, and subjected to paraffin embedding.

### Ethics statement

Our research complies with all relevant ethical regulations. Mouse handling and experimental procedures were approved by the Institutional Animal Care and Use Committee (IACUC) at Emory University in accordance with the US National Institutes of Health Guidelines for the Care and Use of Laboratory Animals and the Animal Welfare Act.

### Pharmacokinetics analyses

PK studies used C57Bl/6 male mice. The compound was formulated for IP administration using 5% v/v NMP + 5% v/v Solutol HS-15 + 90% v/v normal saline to produce a 10 mg/mL solution. PROTAC-6272 was administered at 100 mg/kg IP, and 100 μL of blood was isolated at the indicated time points and separated into plasma. An aliquot of 20 μL plasma was spiked into a 96-well plate, and 200 μL of acetonitrile containing the internal standard was added for protein precipitation. The mixture was vortexed and centrifuged at 4000 rpm for 15 min. 80 μL of the supernatant was transferred into a clean 96-well plate, and 400 μL of H_2_O was added. The mixture was vortexed, and 3.0 µL of the final solution was injected for LC-MS/MS analysis. Separate standard curves were prepared in blank mouse plasma and processed in parallel with the samples. All data were acquired using Analyst 1.7 software (Applied Biosystems). Plasma concentration versus time data were analyzed by non-compartmental approaches using the WinNonlin software program (version 8.3, Pharsight, Mountain View, CA).

### Statistical analysis

One-way ANOVA paired with Tukey’s multiple comparison tests was used to evaluate data consisting of 3 or more groups. Data are presented as mean ± standard error. Significance values were set at *P < 0.05, **P < 0.01, ***P < 0.001. Tumor growth data was analyzed with a two-way ANOVA repeated measures test, combined with Bonferroni’s multiple comparisons test. * Indicates the p value for the comparison between PROTAC-6272 and EPZ-6438, while # indicates the p value for the comparison between PROTAC-6272 and the vehicle group.

## Results

### EZH2 PROTAC design, screening, and efficacy evaluation

**Fig. 1:**
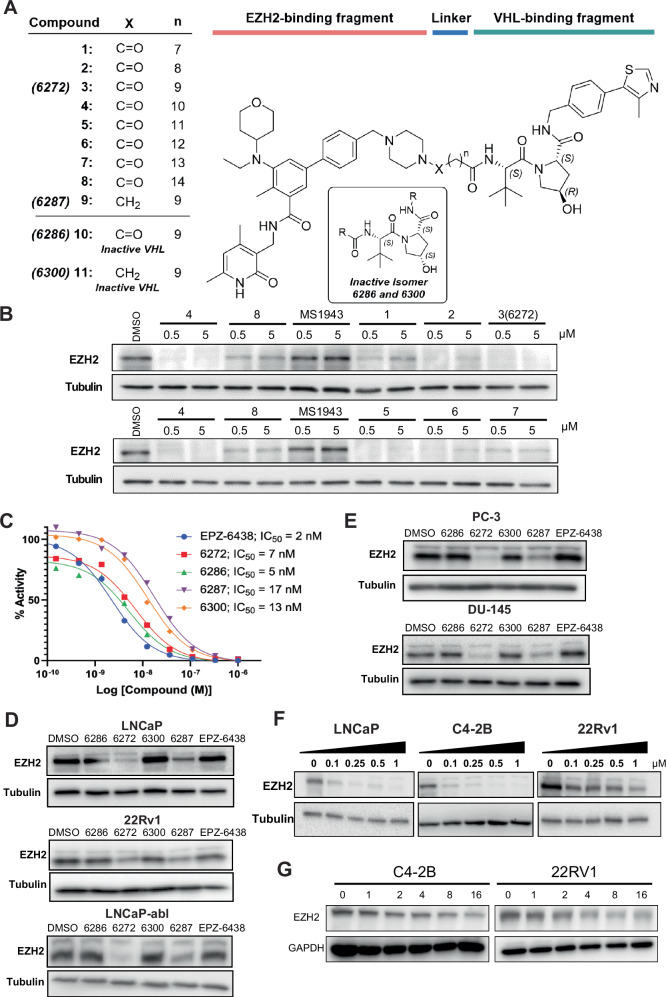
PROTAC design, screening, and efficacy evaluation. **A** Structures of EZH2 PROTACs tested. **B** Screening of PROTAC efficacy in degrading EZH2 compared to DMSO control. C4-2B cells were treated with the indicated concentrations of compounds for 24 h and analyzed by Western blot. **C** Compounds were tested in a radiometric inhibition assay using 5-component PRC2 complex comprising EZH2, EED, SUZ12, RBAP48, and AEBP2 at the indicated concentrations. EPZ-6438 was used as a positive control. **D** AR-positive cell lines LNCaP, 22Rv1, and LNCaP-abl were treated with 0.5 μM of PROTAC-6272 and PROTAC-6287 and their respective negative controls, PROTAC-6286 and PROTAC-6300, for 6 days. Protein lysates were collected and then subjected to Immunoblot analysis. **E** AR-negative cell lines PC-3 and DU-145 were treated with 0.5 μM of PROTAC-6272 and PROTAC-6287 and their respective negative controls, PROTAC-6286 and PROTAC-6300, for 6 days. Protein lysates were collected and then subjected to Immunoblot analysis. **F** LNCaP, C4-2B, and 22Rv1 cells were treated with the indicated doses of PROTAC-6272 for 48 h. Protein lysates were collected and then subjected to immunoblot analysis. **G** C4-2B and 22Rv1 cells were treated with 0.5 μM of PROTAC-6272. Protein lysates were collected at the indicated time course and then subjected to immunoblot analysis.

In pursuit of developing PROTACs targeting EZH2 for degradation, we systematically designed a diverse library of VHL-targeting PROTACs. The FDA-approved EZH2 inhibitor EPZ-6438 was used as the EZH2-targeting ligand based on its potency, selectivity, and desirable safety and pharmacokinetics (PK) properties. A set of VHL-targeting compounds (compounds 1–8), that contained alkyl linkers of increasing length from 7 to 14 methylene units (C7 to C14) were synthesized (Fig. 1A). These compounds were each attached to the EPZ-6438 piperazine group via an amide linkage. We next performed western blot (WB) analyses to determine EZH2 degradation ability of the compounds at 0.5 μM and 5 μM concentration, compared to the DMSO control, in C4-2B PCa cells (Fig. 1B). While all members of this homologous series of alkyl-linked compounds induced significant EZH2 degradation, compound 3 (PROTAC-6272), with a C9 alkyl linker, showed the greatest effect. A gradual increase in degradation was observed going from Compounds 1 to 3 with a C7 to C9 alkyl linkers followed by a gradual decrease in effectiveness going from Compounds 3 to 8 with a C9 to C14 alkyl linker. After identifying C9 as the optimal linker length, we synthesized an analogous compound 9 (PROTAC-6287) with a tertiary amine linkage, which likely exhibits distinct in vivo pharmacokinetics compared to PROTAC-6272 (Fig. 1A).

Based on their superior EZH2 degradation efficiency, PROTAC-6272 and -6287, with potentially different selectivity and PK, were selected for further investigation. We also synthesized corresponding VHL-inactive control compounds, PROTAC-6286 and PROTAC-6300, for PROTAC-6272 and -6287, respectively (Fig. 1A). The control compounds contain an inactive *S*-hydroxypyrrolidine stereoisomer, which renders them unable to bind VHL and thus unable to degrade EZH2. A radiometric inhibition assay using a 5-component PRC2 complex comprising EZH2, EED, SUZ12, RGAP48, and AREBP2 demonstrated the ability of all these PROTACs to potently inhibit the histone methyltransferase activities of PRC2, due to their containing EPZ-6438 (Fig. 1C). PROTAC-6272 and its VHL-inactive control, PROTAC-6286 showed comparable potency to EPZ-6438, but higher potency than PROTAC-6287 and its control, PROTAC-6300, in the methyltransferase assay. We next evaluated the EZH2 degradation efficiency of PROTAC-6272 and PROTAC-6287, compared to their negative controls. A variety of PCa cell lines were treated with 0.5 μM of each compound for 6 days. Both PROTAC-6272 and -6287 efficiently reduced EZH2 levels in androgen receptor (AR)-positive, androgen-sensitive LNCaP and androgen-insensitive 22Rv1 and LNCaP-abl cells, whereas their VHL-inactive controls, PROTAC-6286 and PROTAC-6300, comparable to EPZ-6438, didn’t show EZH2 degradation activity (Fig. 1D). Comparable efficacy was also observed in AR-negative PC-3 and DU-145 PCa cell lines (Fig. 1E). Of note, PROTAC-6272 demonstrated higher EZH2 degradation efficiency than PROTAC-6287 across the tested prostate cell lines and was, therefore, chosen as the compound for further investigation.

To further evaluate the sensitivity and efficacy of PROTAC-6272 in degrading EZH2, we treated LNCaP, C4-2B, and 22Rv1 cells with increasing concentrations (0.1, 0.25, 0.5, and 1 µM) of PROTAC-6272 for 48 h. WB analyses revealed that PROTAC-6272 effectively decreased EZH2 proteins at a concentration as low as 0.1 µM (Fig. 1F). To investigate the kinetics of EZH2 degradation by PROTAC-6272, we treated C4-2B and 22Rv1 cells with 0.5 μM of 6272 and collected protein lysates at different time points (0, 1, 2, 4, 8, and 16 h) for WB analyses. We found that PROTAC-6272 degraded approximately 50% of EZH2 protein as early as 2 h after treatment and showed a time-dependent increase in degradation (Fig. 1G & Fig. S1A-B). Cycloheximide (CHX) chase analysis revealed similar patterns of degradation of EZH2 upon PROTAC-6272 treatment compared with DMSO control, suggesting that this is not due to changes in the rate of transcription (Fig. S1C). Altogether, these results support that our lead PROTAC compound, PROTAC-6272, rapidly and efficiently degrades EZH2 across diverse PCa cell lines.

### PROTAC-6272 selectively degrades PRC2 via VHL-mediated ubiquitination

**Fig. 2:**
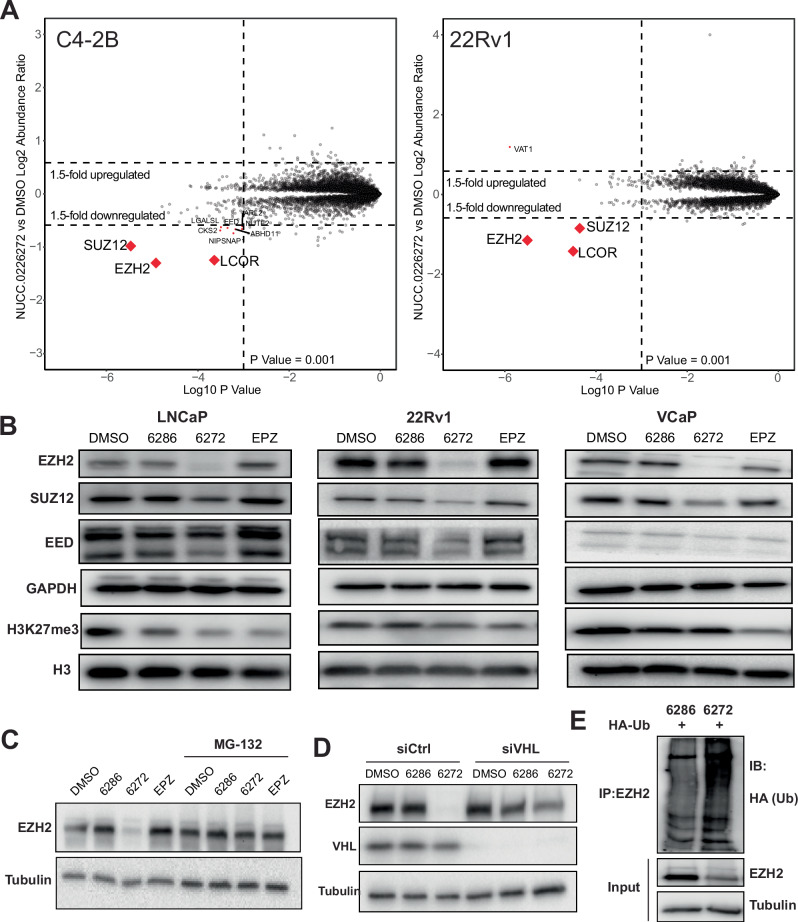
PROTAC-6272 selectively degrades PRC2 via VHL-mediated ubiquitination. **A** Scatter plot of protein expression changes based on global quantitative proteomics. C4-2B cells and 22Rv1 cells were treated with 0.1 µM of PROTAC-6272 for 6 h. Protein lysates were collected and subjected to global quantitative proteomics analysis as described in the experimental section. Data are presented as fold-change normalized to DMSO-treated cells. **B** LNCaP, 22Rv1, and VCaP cells were treated with DMSO, the negative control PROTAC-6286 (0.5 µM), PROTAC-6272 (0.5 µM), and EPZ-6438 (0.5 µM) for 48 h and then subjected to immunoblotting analysis. **C** C4-2B cells were treated with 20 μM proteasome inhibitor (MG-132) for 6 h, then treated with DMSO, negative control PROTAC-6286 (0.1 µM), and PROTAC-6272 (0.1 µM) for 24 h before immunoblotting. **D** C4-2B cells were treated with control (siCtrl) or siVHL for 72 h, followed by treatment with DMSO, negative control PROTAC-6286 (0.1 µM), and PROTAC-6272 (0.1 µM) for 24 h, and then subjected to immunoblotting. **E** C4-2B cells were transfected with HA-Ubiquitin (Ub) for 24 h, then treated with negative control PROTAC-6286 (0.1 µM) and PROTAC-6272 (0.1 µM) for 24 h and subjected to co-IP by anti-EZH2, followed by immunoblotting.

To evaluate the selectivity and specificity of PROTAC-6272, we performed label-free quantitative proteomics in C4-2B and 22Rv1 cells treated with DMSO or 0.1 µM PROTAC-6272 for 6 h. We compared the relative protein abundances in PROTAC-6272-treated cells with those in DMSO-treated controls and identified up- and down-regulated proteins with a threshold of >1.5-fold change and a p value of less than 0.001 (Table S1). Notably, we found that the most significantly downregulated proteins by PROTAC-6272 treatment were PRC2 core subunits, EZH2 and SUZ12, in both C4-2B and 22Rv1 cells (Fig. 2A). In addition, PRC2 accessory protein LCOR (PALI1) [28] was also strongly decreased in both cell lines. On the contrary, EED was less significantly reduced and was reduced only in C4-2B cells, likely due to its PRC2-independent function in interacting with Polycomb Repressive Complex 1 (PRC1) subunits [29]. We then performed WB to validate these proteomics results. WB analyses confirmed that SUZ12 and, to a lesser degree, EED were decreased by 48-h treatment of PROTAC-6272, but not by EPZ-6438 (EPZ) or its VHL-inactive analog PROTAC-6286, in LNCaP, 22Rv1, and VCaP cells (Fig. 2B). As controls, H3K27me3 levels were reduced by all compounds, as they all contained the EPZ-6438 ligand. These findings demonstrated that EZH2 PROTAC degraded not only EZH2 but also other PRC2 complex proteins, likely due to either bystander ubiquitination and degradation caused by the induced proximity of the E3 complex to the protein complex or by destabilizing the PRC2 complex through EZH2 degradation.

We next sought to verify the mechanism of action driving PROTAC-6272-mediated degradation of EZH2. We first evaluated whether this process was dependent on the proteasome. C4-2B cells were pretreated with the proteasome inhibitor MG132 (20 µM) for 6 h and then treated with DMSO, PROTAC-6286, PROTAC-6272, or EPZ-6438 (0.1 µM) for 24 h. We observed that MG132 pretreatment abolished PROTAC-6272-mediated degradation of EZH2 (Fig. 2C). Considering that PROTAC-6272 was designed with a VHL E3-ligase targeting ligand, we next sought to confirm whether PROTAC-6272-mediated degradation of EZH2 depends on binding to VHL. To this end, C4-2B cells were transfected with VHL-targeting siRNA or scrambled control for 72 h, and then treated with DMSO, negative control PROTAC-6286, or PROTAC-6272 at a concentration of 0.1 µM for 24 h. WB demonstrated that EZH2 levels were restored once VHL was knocked down in PROTAC-6272-treated cells, confirming that VHL was, in fact, required for PROTAC-induced EZH2 degradation (Fig. 2D). To further confirm that PROTAC-6272 degraded EZH2 via the VHL-mediated ubiquitin-proteasome system, we examined whether EZH2 ubiquitination is increased upon PROTAC-6272 treatment. C4-2B cells transfected with HA-Ubiquitin (Ub) were treated with PROTAC-6272 or its negative control PROTAC-6286, and co-immunoprecipitation (co-IP) was performed using an EZH2 antibody, followed by immunoblotting with an HA (Ub) antibody. Indeed, we observed an increase in EZH2 ubiquitination upon PROTAC-6272 treatment (Fig. 2E). Taken together, these observations demonstrated that PROTAC-6272 targeted and degraded EZH2 and PRC2 with high selectivity and specificity through the VHL-dependent ubiquitin–proteasome system.

### PROTAC-6272 failed to decrease AR or degrade EZH2 co-activator p300

**Fig. 3:**
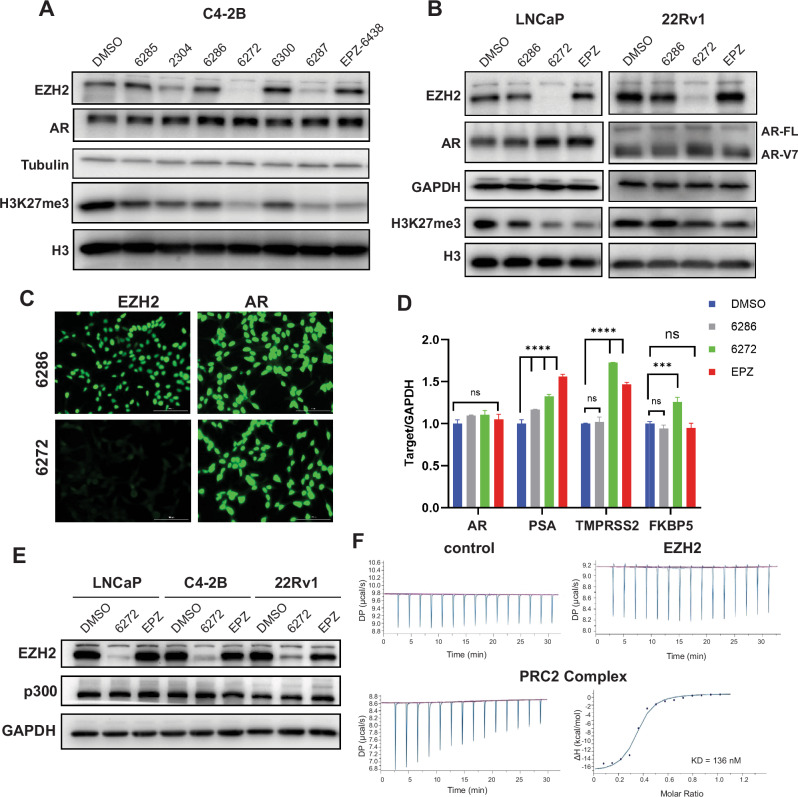
PROTAC-6272 failed to decrease AR transcription or degrade EZH2 co-activator p300. **A** C4-2B cells were treated with DMSO, negative controls PROTAC-6285, -6286, -6300 (0.5 μM), PROTAC-2304, -6272, -6287 (0.5 μM), and EPZ-6438 (0.5 μM) for 72 h. Protein lysates were collected and probed with the indicated antibodies in immunoblotting analysis. **B** LNCaP and 22Rv1 cells were treated with DMSO, negative control PROTAC-6286 (0.5 μM), PROTAC-6272 (0.5 μM), and EPZ6438 (0.5 μM) for 4 days and subjected to immunoblotting analysis. **C** C4-2B cells were treated with 1 μM of negative control PROTAC-6286 or PROTAC-6272 for 72 h, followed by immunofluorescence analysis.d. LNCaP cells were treated with DMSO, negative control PROTAC-6286 (0.5 μM), PROTAC-6272 (0.5 μM), and EPZ6438 (0.5 μM) for 6 days and analyzed by qRT-PCR. Data were normalized to GAPDH. (ns (not significant), ∗∗∗*p* < 0.001, ∗∗∗∗*p* < 0.0001, *n* = 3). **E** LNCaP, C4-2B, and 22Rv1 cells were treated with DMSO, PROTAC-6272 (1 μM), or EPZ-6438 (1 μM) for 2 days, and then subject to immunoblotting analysis. **F** Solo EZH2 protein (85 kDa) and the PRC2 complex (EZH2–EED–SUZ12, VEFS) (150 kDa) were purified from Sf9 insect cells. The interaction of EPZ-6438 with solo EZH2 and the PRC2 complex was tested using Isothermal Titration Calorimetry (ITC).

One key motivation in developing EZH2 PROTACs is to degrade EZH2 protein, thereby targeting both PRC2-dependent and -independent EZH2 functions and being superior to enzymatic EZH2 inhibitors. EZH2, as a transcription coactivator, is well known to interact with p300 [21] and directly induce the transcription of the *AR* gene [14]. However, neither EZH2 coactivators nor AR were found to be significantly altered by EZH2 PROTACs in quantitative proteomics analyses (Fig. 2A). To further investigate this, C4-2B cells were treated with DMSO, EPZ-6438, and various EZH2 PROTACs (-2304, -6272, and -6287) and their negative control (-6285, -6286, -6300), followed by WB analysis. Surprisingly, despite the high efficacy of PROTAC-6272 in degrading EZH2 and reducing H3K27me3, it failed to reduce AR protein (Fig. 3A). Moreover, WB analyses confirmed that EZH2 PROTAC-6272 also failed to decrease AR in LNCaP and 22RV1 cells (Fig. 3B). Immunofluorescent staining further validated that PROTAC-6272 abolished EZH2 staining without affecting AR protein, which localized in the nuclei as expected (Fig. 3C and Fig. S1D). Since EZH2 functions as an activator to induce *AR* gene transcription [14], we performed qRT-PCR analyses. Interestingly, our data revealed that EZH2 PROTAC-6272 did not decrease the mRNA levels of AR (Fig. 3D). In fact, we observed some increase in AR target genes *PSA, TMPRSS2, and FKPB5*, by all compounds, which is consistent with previous reports that AR is also a target of PRC2-mediated epigenetic silencing[14, 30, 31].

We next wondered whether PROTAC-6272 is not able to degrade EZH2 in the co-activator complex. To this end, we performed WB analyses of p300, an EZH2 co-activator and a main enzyme that catalyzes H3K27 acetylation and target gene induction[21]. Notably, we observed no changes in the protein levels of p300 in PCa cells treated with PROTAC-6272 (Fig. 3E). We hypothesized that EZH2 PROTACs derived from enzymatic EZH2 inhibitors, such as EPZ-6438, that binds to the SAM pocket formed by EZH2-EED interaction in the presence of SUZ12 (ref. 9), might be incapable of binding EZH2 outside of the PRC2 complex. To test this, we performed Isothermal Titration Calorimetry (ITC) to measure the ability of EPZ-6438 to bind EZH2 alone (solo EZH2) and EZH2 in the presence of EED and SUZ12 (PRC2-EZH2) (Fig. 3F). Critically, we found that EPZ-6438 bound to PRC2 with a dissociation constant K_D_ = 136 nM ± 53 nM, *N* = 0.55, ΔH = -19.1 ± 1.3 Kcal/mol, and TΔS = -9.73 Kcal/mol. However, it failed to bind to solo EZH2 to any appreciable degree. The result indicates that EPZ-6438 binds specifically to EZH2 within the PRC2 complex but not to solo EZH2. Thus, taken together, these data suggested that PROTAC-6272 failed to decrease AR transcription due to its inability to engage EZH2 outside of the PRC2 complex, such as those associated with coactivator p300, and thus was unable to degrade p300-bound EZH2 or inhibit target gene transcription.

### PROTAC-6272 exhibited superior anti-proliferative activities in some PCa cell lines

**Fig. 4:**
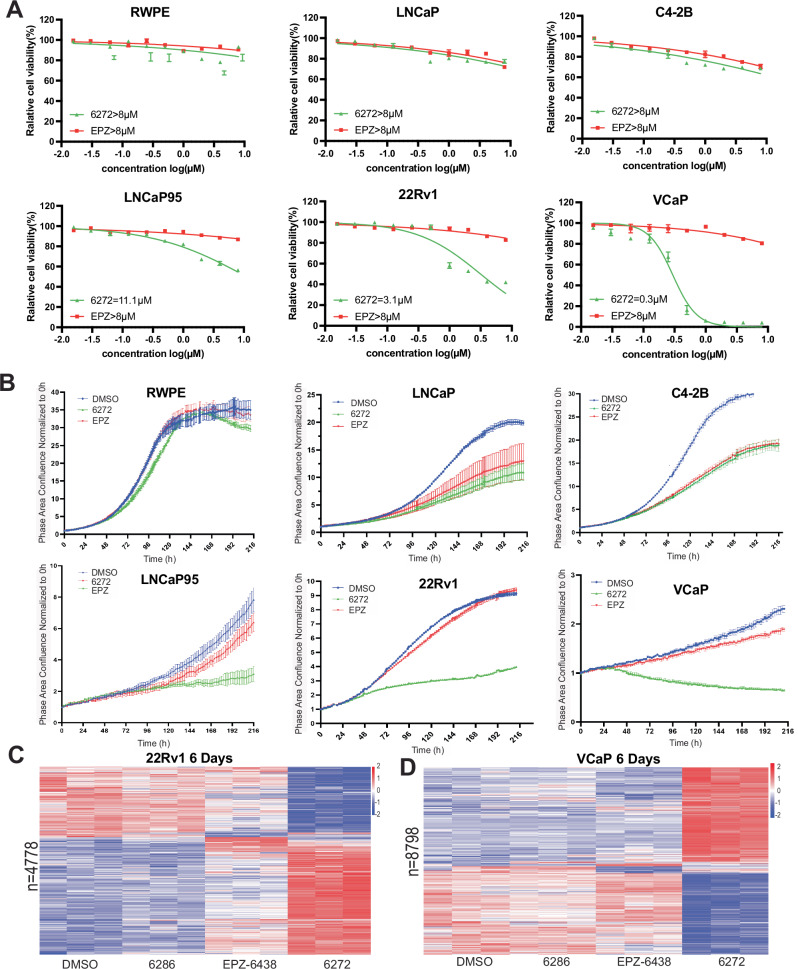
PROTAC-6272 exhibited superior anti-proliferative activity in some PCa cell lines. **A** A panel of PCa cell lines (LNCaP, C4-2B, LNCaP95, 22Rv1, and VCaP) and a normal prostate epithelial cell line (RWPE) were treated with PROTAC-6272 and EPZ-6438 with increasing doses from nM to µM for 48 h, and half inhibitory concentrations (IC_50_) of proliferation rates were assayed by cell Titer Glo (*n* = 6). **B** A panel of PCa cell lines was treated with DMSO, PROTAC-6272 (1 µM), and EPZ-6438 (1 µM) and incubated in an IncuCyte live imager chamber; images were taken every 2 h up to 9 days. Cell proliferation rates were analyzed by IncuCyte live imager software (*n* = 6). **C** 22Rv1 cells were subjected to triplicate RNA-seq analysis upon 6 days of treatment with DMSO, PROTAC-6286, PROTAC-6272, and EPZ-6438 (1 µM). Heatmap shows combined differentially expressed genes identified ( | Log2FC | ≥0.585, adjusted *p* < 0.05) from treatment of PROTAC-6286, EPZ-6438, or PROTAC-6272 relative to DMSO. **D** VCaP cells were subjected to triplicate RNA-seq analysis upon 6 days of treatment with DMSO, PROTAC-6286, PROTAC-6272, and EPZ-6438. Heatmap shows combined differentially expressed genes identified ( | Log2FC | ≥0.585, adjusted *p* < 0.05) from treatment of PROTAC-6286, EPZ-6438, or PROTAC-6272 relative to DMSO.

We have thus far found that, albeit unable to inhibit EZH2 activator function, EZH2 PROTACs effectively degraded EZH2 and PRC2, which might still render better efficacy than the inhibition of EZH2 enzymatic activities only. We first assessed the cell viability of a panel of human PCa cell lines, along with a benign prostate epithelial cell line, in response to increasing doses of PROTAC-6272, compared to EPZ-6438, for 48 h. Exposure to PROTAC-6272 did not affect cell proliferation in the benign prostate epithelial cell line RWPE, indicating that PROTAC-6272 had no major toxicity (Fig. 4A). PROTAC-6272 also showed minimal effects on the viability of LNCaP and C4-2B cells and exhibited no significant difference compared to EPZ-6438. Notably, PROTAC-6272 effectively suppressed cell proliferation in LNCaP95, 22Rv1, and VCaP cells, with IC_50_ values ranging from 0.3 to 11.1 μM. In contrast, EPZ-6438 remained ineffective in suppressing these cells (Fig. 4A). These results indicated that, following short-term treatments, LNCaP95, 22Rv1, and VCaP cells are more sensitive to PROTAC-6272 than LNCaP and C4-2B cells, and that PROTAC-6272 is more effective at reducing cell proliferation than the EZH2 inhibitor EPZ-6438.

To further validate these interesting cell-type-dependent effects, we utilized the IncuCyte® live cell analysis system to monitor long-term effects on cell proliferation. Each cell line was individually seeded in a 96-well plate and then imaged every 2 h, up to 9 days, in the IncuCyte live imager chamber. In line with the short-term results, PROTAC-6272 exhibited a much stronger growth inhibitory effect than EPZ-6438 in LNCaP95, 22Rv1, and VCaP cells, but showed a modest efficacy comparable to EPZ-6438 in LNCaP and C4-2B cells, while no growth inhibitory effects were observed in RWPE benign prostate cells (Fig. 4B). As controls, we performed EZH2 knockdown using shRNA and found that it abolished both 22Rv1 and C4-2B cell proliferation (Fig. S2), indicating multifaceted roles of EZH2 in PCa cells that were only partially targeted by the compounds, even PROTACs. Furthermore, we performed colony formation assays for LNCaP, C4-2B, VCaP, and 22Rv1 cells (Fig. S3A), and the results were largely consistent with the cell proliferation data.

RNA-seq analyses of PCa cells treated with the EZH2-targeting compounds for 6 days revealed a much stronger effect of PROTAC-6272, compared to EPZ-6438, in regulating target gene expression in VCaP and, to a lesser extent of difference, 22Rv1 cells (Fig. 4C, D, Table S2, S3), while comparable effects were observed in LNCaP (Fig. S3B, Table S4). These molecular changes are consistent with their respective anti-growth effects. Further, Gene Ontology (**GO**) analyses revealed that PROTAC-6272 inhibited genes involved in chromosome segregation, cell cycle, and cytoplasmic translation (Fig. S3C–E). Interestingly, neither PROTAC-6272 nor EPZ-6438 inhibited the growth of AR-negative PC3 cells, despite the ability of PROTAC-6272 to degrade EZH2 (Fig. S4). In contrast, previous studies have demonstrated that EZH2 knockdown abolished AR-negative PC3 and DU145 cell growth and motility [32, 33]. Taken together, these findings indicate that PROTAC-6272 exhibits greater anti-proliferative efficacy than the enzymatic inhibitor EPZ-6438 in some prostate cancer cell lines, but it remains unable to eliminate all functions of EZH2.

### PROTAC-6272 rapidly induces cellular senescence independently of methylation

**Fig. 5:**
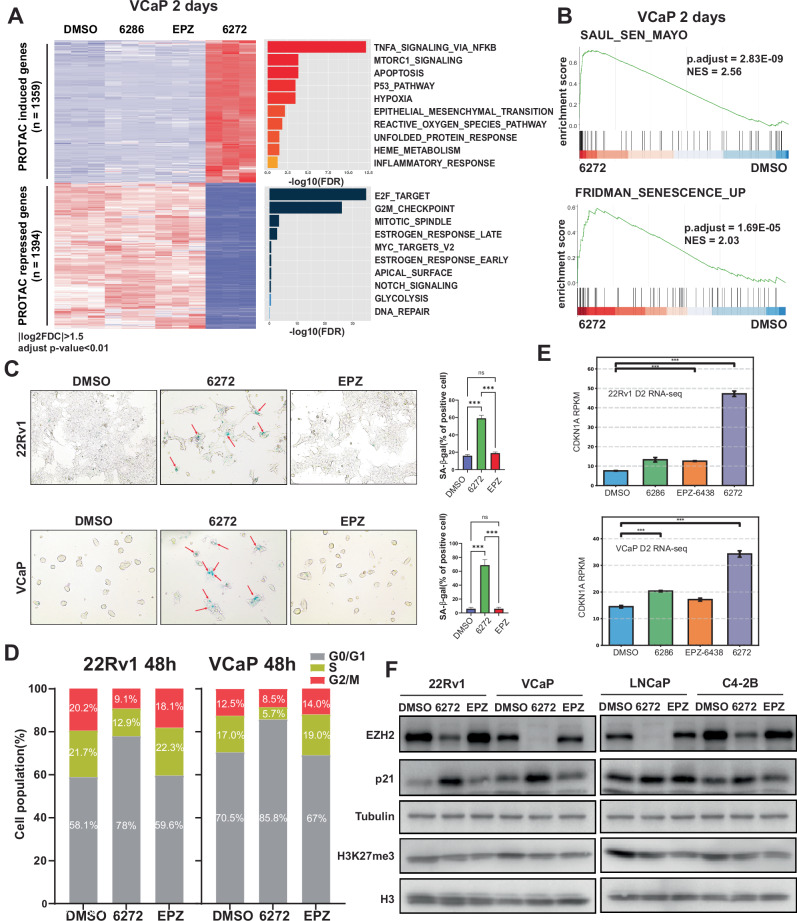
PROTAC-6272 induces cellular senescence independently of methylation. **A** VCaP cells were subjected to triplicate RNA-seq analysis upon 48 h of treatment with DMSO, PROTAC-6286, PROTAC-6272, and EPZ-6438 (1 µM). Heatmap shows differentially expressed genes regulated by PROTAC-6272. Gene ontology (GO) analysis reveals the top 10 HALLMARK concepts induced and repressed by PROTAC-6272. **B** Gene Set Enrichment Analysis (GSEA) was performed to determine the enrichment of two public cellular senescence gene sets, SAUL_SEN_MAYO (*n* = 124) and FRIDMAN_SENESCENCE_UP (*n* = 77), in the gene expression dataset of VCaP cells treated with DMSO or PROTAC-6272. **C** SA-β-gal staining to test cell senescence in 22Rv1 and VCaP cells treated with 1 µM of PROTAC-6272 for 48 h. SA-β-Gal positive cells (red arrows) were displayed in light blue and quantified as a percentage of total cells in randomly selected fields (ns, not significant;, ∗∗∗*p* < 0.001, *n* = 6). **D** Flow cytometry analysis of cell cycle progression in 22Rv1 and VCaP cells. **E** 22Rv1 and VCaP cells were treated with DMSO, PROTAC-6272, and EPZ for 48 h. CDKN1A (p21) expression in each cell line was quantified from RNA-seq data using RPKM (***p < 0.001, *n* = 3). **F** 22Rv1 and VCaP cells were treated with DMSO, PROTAC-6272, and EPZ (1 µM) for 48 h. Protein lysates were collected and probed with the indicated antibodies in immunoblotting analysis.

We next attempted to further explore the molecular mechanism underlying the enhanced efficacy and anti-proliferative activity of PROTAC-6272 in some PCa cell lines. To capture potential targeting of non-catalytic functions of EZH2, we treated VCaP cells with DMSO, PROTAC-6286, PROTAC-6272, or EPZ-6438 for a short period (48 h), followed by RNA-seq. RNA-seq analysis identified 1359 up-regulated and 1394 down-regulated genes (adjusted p value < 0.01 and |log2 fold-change | > 1.5) in 6272-treated relative to DMSO-treated cells. Critically, these genes were not differentially regulated by EPZ-6438 or the VHL-inactive control compound PROTAC-6286, suggesting that their regulation is due to EZH2 degradation rather than epigenetic regulation (Fig. 5A, Table S5). Pathway enrichment analysis revealed that PROTAC-6272-induced genes were strongly enriched for pathways related to proinflammatory responses, such as tumor necrosis factor alpha (TNF-α) signaling via NF-κB, while PROTAC-6272-repressed genes were involved in cell cycle regulation such as E2F target and G2M checkpoint. These PROTAC-6272-specific effects were also observed in 22Rv1 cells treated with the compounds for 2 days (Fig. S5A, Table S6). Notably, previous studies have reported that the induction of pro-inflammatory genes and repression of cell cycle-related genes are suggestive of the onset of cellular senescence [34].

To confirm this, we performed gene set enrichment analyses (GSEA) to determine whether gene sets related to pro-inflammatory senescence-associated secretory phenotype proteins (SASPs) were altered in PROTAC-6272-treated cells. We used two well-established SASPs human gene sets, “SAUL_SEN_MAYO” [35] and “FRIDMAN_SENESCENCE_UP” [36], for the analysis. GSEA showed that both SASPs were significantly up-regulated in PROTAC-6272-treated compared to control in VCaP and 22Rv1 cells (Fig. 5B and Fig. S5B). To further confirm whether PROTAC-6272 induces cellular senescence, we stained senescence-associated β-galactosidase (SA-β-Gal) in 22Rv1 and VCaP cells following DMSO, PROTAC-6272, and EPZ-6438 treatment for 48 h. SA-β-Gal-positive cells were displayed in light blue and scored as a percentage of total cells in several randomly selected fields. Importantly, PROTAC-6272 induced SA-β-Gal staining in both 22Rv1 and VCaP cells, indicative of cellular senescence, while EPZ-6438 showed no apparent effects (Fig. 5C).

Cellular senescence is a stable cell cycle arrest pathway that can be triggered in normal cells in response to various intrinsic and extrinsic stimuli [37]. It is characterized by cell-cycle arrest in the G_1_ or possibly G2 phase to prevent the proliferation of damaged cells [38–40]. Therefore, to test whether PROTAC-6272 causes cell-cycle arrest via cellular senescence, we performed flow cytometry analysis of cell cycle progression in 22Rv1 and VCaP cells. Cells were treated with DMSO, PROTAC-6272, and EPZ-6438 for 48 h, followed by fixation and DAPI staining. Significantly, flow cytometry analyses indicated that PROTAC-6272 treatment indeed induced G0/G1 phase cell arrest, whereas EPZ-6438 did not alter cell cycle as compared to DMSO control cells (Fig. 5D).

Growth arrest during cellular senescence is initiated and enforced by the cyclin-dependent kinase and cell cycle inhibitors p16 and p21 (encoded by the *CDKN2A* and *CDKN1A* genes, respectively) [41]. EZH2 knockdown has previously been shown to lead to rapid cellular senescence via p21 upregulation, which is dependent on DNA replication, but independent of H3K27 methylation [42]. This is in contrast to methylation-dependent p16 up-regulation, which takes a longer time to occur. Indeed, RNA-seq data revealed a stronger upregulation of *CDKN1A* (p21) transcription after 48 h of PROTAC-6272 treatment compared to EPZ-6438, in 22Rv1 and VCaP cells, but not in LNCaP cells (Fig. 5E, Fig. S5C–E, Table S7). Furthermore, WB analyses confirmed an increase in p21 protein in VCaP and 22Rv1 cells treated with PROTAC-6272, but not EPZ-6438, despite their similar effects in reducing H3K27me3 levels. Critically, this superiority of PROTAC 6272 is diminished in LNCaP and C4-2B cells (Fig. 5F), consistent with the absence of a superior growth-inhibitory effect in these cells (Fig. 4A, B). We next treated 22Rv1 cells with PROTAC-6272 and performed immunofluorescent (IF) co-staining of PCNA and 53BP1, which respectively measure DNA replication and DNA double-strand breaks, key triggers of the senescence program. Importantly, our results revealed 53BP1 foci frequently colocalized with sites of PCNA labeling after the treatment of PROTAC-6272 (Fig. S6). Cellular senescence is characterized by the irreversible arrest of the cell cycle, which can ultimately lead to apoptosis. To evaluate apoptosis, we performed caspase-3/7 fluorescence staining in VCaP cells treated with DMSO, PROTAC-6272, or EPZ-6438. A significantly higher level of apoptosis was observed in the PROTAC-6272 treatment group compared with the controls (Fig. S7). These results indicated that PROTAC-6272, but not EPZ-6438, rapidly increased p21 expression and, thus, cellular senescence in some PCa cells through methylation-independent but DNA replication-dependent mechanisms.

### PROTAC-6272 inhibited VCaP xenograft tumor growth in vivo

**Fig. 6:**
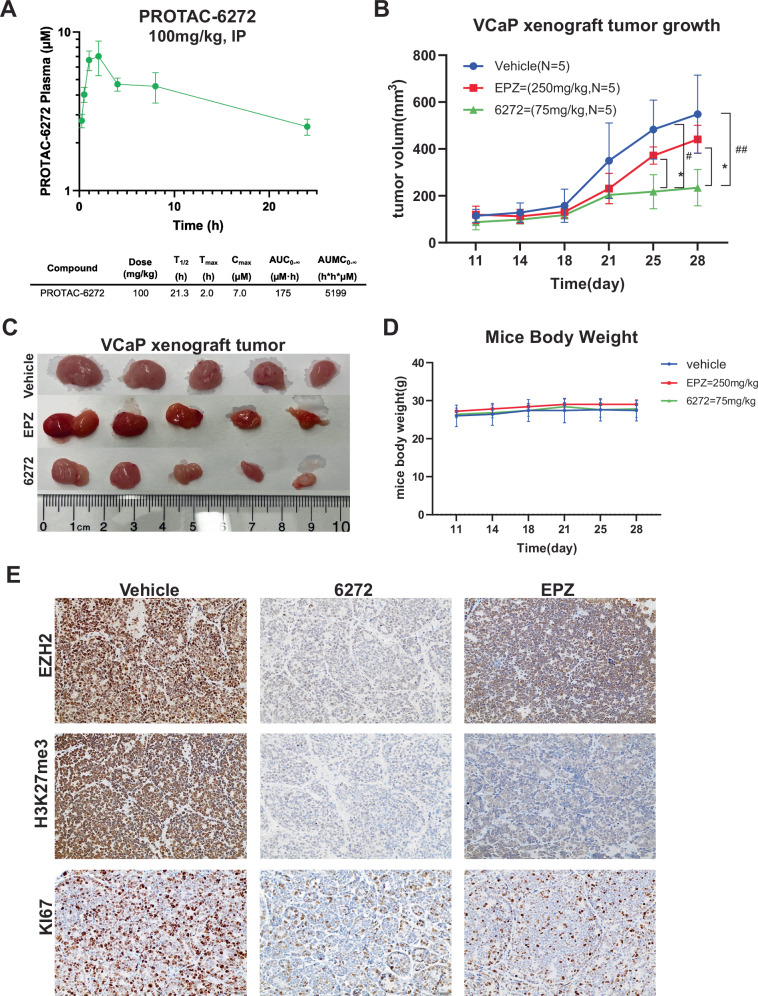
PROTAC-6272 inhibited VCaP xenograft tumor growth in vivo. **A** Pharmacokinetics of PROTAC-6272 in C57BL/6 mice. The compound was dosed at 100 mg/kg by IP. **B** VCaP cells were injected subcutaneously into the right flanks of NGS mice. After 11 days post inoculation, mice were treated with either vehicle (*n* = 5), PROTAC-6272 (*n* = 5, 75 mg/kg), or EPZ-6438 (*n* = 5, 250 mg/kg) for 18 days. Tumor growth data are shown as mean ± SD. (*indicates the pvalue for the comparison between PROTAC-6272 and EPZ-6438 (∗*p* < 0.05, ∗∗*p* < 0.01), while ^#^indicates the pvalue for the comparison between PROTAC-6272 and the vehicle group (^#^*p* < 0.05, ^##^*p* < 0.01), two-way ANOVA repeated measures test, combined with Bonferroni’s multiple comparisons test). **C** Gross images of treated tumors were collected at the endpoint of the study. **D** Body weights of the mice undergoing the indicated treatments (*n* = 5). **E** Representative IHC images of tumor sections stained for EZH2, H3K27me3, and KI67.

Finally, to evaluate the therapeutic potential of PROTAC-6272 in vivo, we evaluated its efficacy and PK properties. We first carried out PK studies of PROTAC-6272 to determine whether sufficient plasma exposure can be obtained to produce the needed in vivo efficacy. After a single 100 mg/kg dose administered intraperitoneally (IP), plasma was analyzed at sequential time points and quantified by LC/MS/MS. Importantly, PROTAC-6272 was found to have a long half-life of 21.3 h, with a C_max_ = 7.0 μM (Fig. 6A). Total exposure (AUC_0-∞_) was 175 μM-hr, and plasma protein binding was 99.8%, supporting feasibility for in vivo use.

Next, we sought to test the efficacy of PROTAC-6272 in inhibiting PCa growth in vivo. Immune-deficient mice were subcutaneously inoculated with VCaP cells, based on their high sensitivity to PROTAC-6272 in vitro. At eleven days post-inoculation, tumors were palpable, and mice began to receive treatment with vehicle control (*N* = 5), PROTAC-6272 (*N* = 5, 75 mg/kg), or EPZ-6438 (*N* = 5, 250 mg/kg) for 18 days. Tumor volumes were monitored every 2–3 days. Importantly, while EPZ-6438 was able to slightly suppress VCaP xenograft tumor growth compared to vehicle control, PROTAC-6272 demonstrated significantly greater inhibition than EPZ-6438, nearly flattening the tumor growth curve (Fig. 6B). Consistently, endpoint tumors in the PROTAC-6272-treated group were substantially smaller compared to those in the EPZ-6438 and vehicle control groups (Fig. 6C). Importantly, no significant changes in body weight were observed for mice treated with PROTAC-6272 and EZP-6438 relative to vehicle controls, suggesting that the drugs were tolerable to the mice (Fig. 6D). Lastly, to confirm the on-target effect of PROTAC-6272, tumor sections were stained for EZH2, H3K27me3, and Ki67. Our results showed that EZH2 was eliminated in tumors treated by PROTAC-6272, but not EPZ-6438, whereas H3K27me3 was abolished by both compounds, being consistent with their respective mechanisms of action (Fig. 6E). Of note, Ki67 staining confirmed markedly reduced rates of cell proliferation in tumors treated by PROTAC-6272, and to a lesser extent, by EPZ-6438. Taken together, these findings show that PROTAC-6272 is well tolerated and exhibits greater efficacy than the EZH2 inhibitor EPZ-6438 in inhibiting VCaP xenograft tumor growth in vivo.

## Discussion

EZH2 has been shown as a major oncogene and a therapeutic target in various cancers for over two decades, and numerous EZH2 inhibitors have been developed and tested in preclinical models and clinical trials [8, 43, 44]. Unfortunately, a vast majority of these, including EPZ-6438, are EZH2 catalytic inhibitors and have shown poor efficacy against PCa, even though EZH2 is frequently overexpressed in advanced PCa. Recent evidence emerged that EZH2 has non-catalytic functions that are insensitive to its enzymatic inhibitors [14, 45, 46]. For instance, we have previously shown that EZH2 induces AR gene transcription independently of its methyltransferase activity. Such findings suggested that complete depletion of EZH2 might be necessary in abolishing all EZH2 functions, which led to the rapid development of many EZH2-degrading PROTACs [19, 20, 23, 26]. Surprisingly, EZH2 degraders continued to exhibit varying growth-inhibitory efficacy, depending on the degrader and the cell models tested. Attempting to solve such puzzles, we developed an EZH2 degrader, PROTAC-6272. We showed that it targets EZH2 and PRC2 core subunits SUZ12 and EED for degradation, as well as PRC2 accessory proteins, in an exquisitely selective manner.

However, to our surprise, PROTAC-6272 failed to decrease the transcription of EZH2 target gene AR. Mechanistically, we found that PROTAC-6272 was unable to degrade p300, a critical co-activator of EZH2 that catalyzes H3K27 acetylation and is necessary for the active transcription of target genes, including AR. This led us to hypothesize that PROTACs that were derived from enzymatic EZH2 inhibitors might not be able to engage the EZH2 proteins in co-activator complexes. This was supported by our ITC data, which showed that EPZ-6438 can only interact with EZH2 in the presence of other core subunits, SUZ12 and EED, but not with solo EZH2. It is well known that EZH2 minimally requires EED and SUZ12 for its catalytic activity. One likely reason is that EED and SUZ12 help reshape the EZH2 catalytic domain to allow binding of the histone substrate and SAM [47, 48], and likewise EPZ-6438. Indeed, while a small portion of EPZ-6438 interacts with the SAM-binding pocket of the active enzyme complex, most of the molecule binds to an extended loop surface that appears to be available only when the EZH2–EED–SUZ12 ternary complex is formed [49]. Therefore, it is not surprising that EPZ-6438 shows no detectable binding to solo EZH2 by ITC, in contrast to the active PRC2 complex. These findings are highly significant as they revealed a critical mechanism underlying the lack of efficacy data of many previously reported EZH2 PROTACs in inhibiting PCa cells [19, 20, 23, 26]. This data also suggests that combining EZH2 PROTAC with an AR-targeting agent, such as enzalutamide, a MYC inhibitor, or a p300 inhibitor, may be beneficial to advanced PCa patients.

Nevertheless, PROTAC-6272 exhibited much stronger anti-proliferative activity than EPZ-6438 for some PCa lines, including LNCaP95, 22Rv1, and VCaP. Such improved efficacy of PROTAC-6272 is likely due to its rapid induction of cellular senescence. Cellular senescence, first observed by Hayflick and Moorhead in human diploid fibroblasts [50], is a state of irreversible cell cycle arrest regulated primarily by CDKN1A (p21) and CDKN2A (p16). We found that PROTAC-6272 rapidly induced p21, which appears to be independent of H3K27 methylation, in LNCaP95, 22Rv1, and VCaP cells. PROTAC-6272 showed no superiority in growth inhibition over EPZ-6438 in LNCaP and C4-2B cells (Fig. 4), wherein it failed to induce p21 expression. Of note, when these cells are plated at low density, as in the colony formation assays (Figure. S3), PROTAC-6272 exhibited slightly stronger growth inhibition than EPZ-6438. This is likely because the cellular senescence program is suppressed by cell-cell contact inhibition and high cell density [51, 52]. Cells do not undergo senescence when under cell cycle arrest due to contact inhibition [52]. Thus, EZH2-PRC2 represses p21 through methylation-independent mechanisms in some PCa cells, rendering their selected sensitivity to PROTAC-6272, but not enzymatic EZH2 inhibitors such as EPZ-6438. EZH2 protein (within PRC2) has an important structural function at the replication fork. Without PRC2 complex binding at the replication fork, the cell undergoes cell cycle arrest and rapidly triggers cell senescence, leading to apoptosis independently of its catalytic function. Our results are consistent with previous studies indicating that this process is dependent on DNA replication [53]. However, our data does not exclude the possibility of cell-type-specific effects of EZH2-PRC2 in regulating other pathways, which is beyond the scope of the current study. Despite its limitations, PROTAC-6272 exhibits efficient EZH2 and PRC2 degradation, potent anti-tumor activity in some PCa, favorable pharmacokinetic properties, and low toxicity, highlighting the therapeutic potential of EZH2 PROTACs in certain PCa contexts. Moreover, given that EZH2 overexpression is a major oncogenic driver in several cancers, PROTAC-6272 may have broader applications in other EZH2-overexpressing or -activated cancers, warranting further exploration.

## Supplementary information

supp figures methods

supp table

## Data Availability

All next-generation sequencing data have been uploaded to the GEO database under the GSE number: GSE298094.
