# Supplementary material for: EZH2 PROTACs outperform catalytic inhibitors in prostate cancer by targeting a methylation-independent function of PRC2
Source: Oncogene. 2026 Jan 7;45(5):636–49. doi: 10.1038/s41388-025-03662-z (PMC12846922; doi:10.1038/s41388-025-03662-z)
Supplement: Supplementary file 1 — supp figures methods [file 41388_2025_3662_MOESM1_ESM.pdf]

Supplementary Information

EZH2 PROTACs Outperform Catalytic Inhibitors in Prostate Cancer by Targeting a Methylation-Independent Function of PRC2

Wanqing Xie,[1] Qi Chu,[1] Lourdes Brea,[1] Guihua, Zeng,[1] Yuan Wang[1], Xiaodong Lu,[1] Mohan Zheng[1], Corinne R. Ley,[2] Zhiquan Lei,[2] Hongshun Shi,[1] Joshua L. Zhu,[2] Lihu Gong,[3] M. Cynthia Martin,[2] Xianglin Shi,[1] Galina Gritsina,[1] Arabela A. Grigorescu,[4] Hana Chandonnet,[1] Xin Liu,[3] Jonathan C. Zhao,[6,7] Gary E. Schiltz,[2,5,8] Jindan Yu[1,6,7]

Supplementary Methods:
Method S1: Synthesis of Compound 6272 and 6286
Method S2: diaPASEF quantitative LCMS proteomics and data analysis

Supplementary Figures:
Figure S1. EZH2 degradation by PROTAC-6272
Figure S2. IncuCyte Cell proliferation assay to test EZH2 knockdown in C4-2B and 22Rv1 cells
Figure S3. Colony formation and RNA-seq analysis of various PCa cell lines
Figure S4. Effect of PROTAC-6272 in AR-negative cells
Figure S5. RNA-seq data of PCa cells
Figure S6. PCNA and 53BP1 IF staining in VCaP cells
Figure S7. Caspase3/7 fluorescence staining to test apoptosis in VCaP cells

Supplementary Tables:
Table S1: Quantitative proteomics analysis of PROTAC-6272 treated C4-2B and 22Rv1 cells
Table S2: Differentially expressed genes in 22RV1 cells treated for 6 days, related to Fig 4C.
Table S3: Differentially expressed genes in VCaP cells treated for 6 days, related to Fig 4D.
Table S4: Differentially expressed genes in LNCaP cells treated for 6 days, related to Fig S3B.
Table S5: Differentially expressed genes in VCaP cells treated for 2 days, related to Fig 5A.
Table S6: Differentially expressed genes in 22RV1 cells treated for 2 days, related to Fig S5A.
Table S7: Differentially expressed genes in LNCaP cells treated for 2 days, related to Fig S5C.

Supplementary References

Supplementary Methods

Method S1: Synthesis of Compound 6272 and 6286

Unless otherwise noted, all purchased reagents were used as received without further purification. Reagent **1** was prepared according to literature methods [1,2] Normal-phase column chromatography was performed using silica gel columns and ACS grade solvents. ^{1}H NMR and ^{13}C NMR spectroscopy were recorded on Bruker 400 MHz or Bruker Avance III 500 MHz spectrometers. The chemical shifts for ^{1}H NMR are reported to the second decimal place in parts per million (ppm). Proton coupling constants are expressed in hertz (Hz). Standard abbreviations were used to denote spin multiplicity for ^{1}H NMR data. The chemical shifts for ^{13}C NMR are reported to the first decimal place in ppm. The representative residual solvent peaks ($CDCl_3$,^{1}H δ = 7.27 ppm, ^{13}C δ = 77.16 ppm; CD3OD-d_4, ^{1}H δ = 3.31 ppm, ^{13}C δ = 49.00 ppm; DMSO-d_6, ^{1}H δ = 2.50 ppm, ^{13}C δ = 39.52 ppm) were used as an internal standard. High-resolution mass spectrometry (HRMS) values were measured and calculated with an Agilent 6545 QTOF mass spectrometer coupled with an Agilent 1200 series LC, with direct loop injection (no column). Low-resolution liquid chromatography/mass spectrometry (LCMS) was performed on a Waters Acquity-H UPLC/MS system with a 2.1 mm × 50 mm, 1.7 µm, reversed phase BEH C18 column and LCMS grade solvents. Gradient elution from 95% water +0.1% TFA/5% acetonitrile +0.1% TFA to 95% acetonitrile +0.1% TFA/5% water +0.1% TFA over 2 min plus a further minute continuing this mixture at a flow rate of 0.85 mL/min was used as the eluent. Total ion current traces were obtained for electrospray positive and negative ionization (ESI^{+}/ESI^{-}). All compounds presented in the manuscript are >95% pure by HPLC analysis.

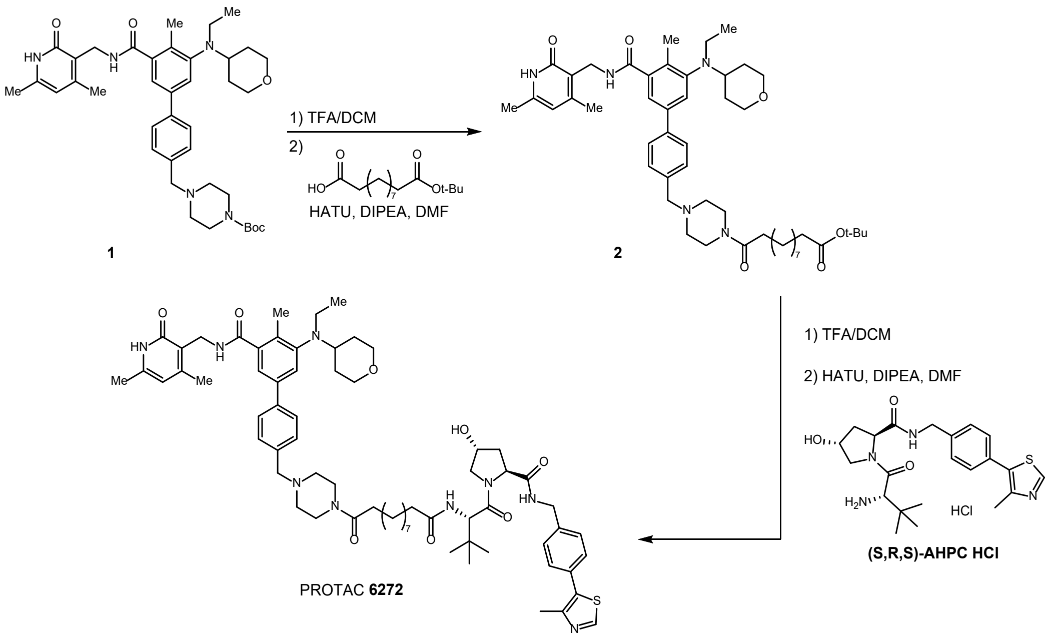

Synthetic scheme for PROTAC 6272

To a 100 mL flask with tert-butyl 4-((3'-(ethyl(tetrahydro-2H-pyran-4-yl)amino)-4'-methyl-5'-(((6-methyl-2-oxo-1,2-dihydropyridin-3-yl)methyl)carbamoyl)-[1,1'-biphenyl]-4-yl)methyl)piperazine-1-carboxylate **1** (4.1 g, 5.42 mmol) and DCM (100 ml) was added TFA (10.8 mL, 26 equiv, 141 mmol) and stirred at 0 ºC for 10 min, then warmed to room temperature over 2.5 hours. The solvent and excess TFA were removed *in vacuo*, then the residue was redissolved in DCM and reconcentrated under a stream of N_2 to remove residual TFA.

To a 100 mL flask was added 11-(tert-butoxy)-11-oxoundecanoic acid (1.55 g. 1.05 eq), DMF (30 mL), followed by DIPEA (3.78 mL, 4 equiv, 21.7 mmol) and HATU (2.16 g, 1.05 equiv, 5.69 mmol) and stirred at room temperature for 30 min. This mixture was added to the deprotected material from above (1.0 eq, 5.42 mmol) and stirred at room temperature overnight. The reaction mixture was diluted with saturated $NaHCO_3$ solution (40 mL), extracted with EtOAc (2 x 70 mL), then extracted by DCM (2 x 40 mL). The combined EtOAc layers were washed with brine (2 x 20 mL), then the combined DCM layers were washed with brine (2 x 20 mL). All the organic phases were combined, dried with Na_2SO_4, filtered, concentrated to dryness, and purified via flash column chromatography (0-5% MeOH/DCM) to give the intermediate **2**, which was carried forward without additional purification.

To a 100 mL flask was added intermediate **2** (4.5g, 3.51 mmol) and DCM (36 ml). TFA (8.4 mL, 30 equiv) was added at room temperature and the mixture stirred overnight. The solvent and excess TFA was removed *in vacuo*, then redissolved in DCM and concentrated under a stream of N_2 to give a dark red oil that was used without further purification. This red oil was dissolved in DMF (30 mL), then DIPEA (9.2 mL, 15 equiv, 52.8 mmol) and HATU (1.34 g, 1.0 equiv, 3.6 mmol) were added. The mixture was stirred at room temperature for 30 min, then (*S,R,S*)-AHPC hydrochloride was added in one portion and the mixture was stirred at room temperature overnight. Saturated $NaHCO_3$ solution (60 mL) was added and the solution extracted with DCM (3x 90 mL). The combined organic layers were washed with saturated NaCl (3 x 30 mL), dried with Na_2SO_4, filtered, and concentrated. The residue was purified via flash column chromatography (5-10% MeOH/DCM w/0.1% HCO_2H) to give PROTAC **6272** mixed with its formate ester. This material was dissolved in MeOH (70 mL) and K_2CO_3 (1.9 g, 4 equiv, 14 mmol) was added. The mixture was stirred at 36 ºC for 4 hours, at which point it was concentrated *in vacuo*, diluted with water and extracted 3x with DCM (3 x 50 mL). The combined organics were dried with Na_2SO_4, filtered, and concentrated to afford PROTAC **6272** as a white solid (2.2 g, 52%). ^{1}H-NMR (500 MHz, CD_3OD) δ 8.88 (s, 1H), 7.57 (d, J = 8.2 Hz, 2H), 7.48 (d, J = 8.2 Hz, 3H), 7.45 – 7.37 (m, 4H), 7.34 (d, J = 1.6 Hz, 1H), 6.13 (s, 1H), 4.66 (s, 1H), 4.62 – 4.53 (m, 2H), 4.51 (s, 3H), 4.37 (d, J = 15.5 Hz, 1H), 3.93 (t, J = 10.2 Hz, 3H), 3.82 (dd, J = 10.9, 3.9 Hz, 1H), 3.58 (d, J = 14.3 Hz, 6H), 3.42 – 3.35 (m, 2H), 3.17 (d, J = 7.0 Hz, 3H), 2.49 (d, J = 4.6 Hz, 5H), 2.47 – 2.43 (m, 2H), 2.41 (s, 3H), 2.40 – 2.36 (m, 2H), 2.35 (s, 3H), 2.33 – 2.19 (m, 6H), 2.10 (ddd, J = 13.3, 9.1, 4.5 Hz, 1H), 1.77 (d, J = 11.3 Hz, 2H), 1.63 (ddd, J = 32.6, 13.3, 5.5 Hz, 6H), 1.33 (s, 12H), 1.05 (s, 9H), 0.92 (t, J = 7.0 Hz, 3H). ^{13}C NMR (126 MHz, CD_3OD) δ 175.95, 174.38, 174.02, 172.88, 172.31,

165.57, 153.42, 152.77, 150.67, 148.98, 144.82, 140.73, 140.48, 140.26, 139.63, 137.78, 134.39, 133.34, 131.46, 131.08, 130.30, 128.96, 127.77, 125.19, 122.73, 122.14, 111.00, 71.04, 68.22, 63.28, 60.80, 59.73, 58.90, 58.02, 43.67, 43.10, 42.61, 38.92, 36.63, 36.58, 33.96, 31.85, 30.43, 30.39, 30.36, 30.33, 30.22, 27.08, 26.99, 26.55, 19.77, 18.65, 15.89, 15.16, 13.16. HRMS (ESI+): m/z calcd for $C_{67}H_{91}N_9NaO_8S$: 1204.6604 $[M+Na]^+$; found: 1204.6609 $[M+Na]^+$.

PROTAC **6286** was prepared analogously, starting from intermediate **2**: To intermediate **2** (250 mg, 1 equiv, 303 μmol) in DCM (5 mL) was added TFA (699 μL, 30 equiv, 9.08 mmol) and the reaction was stirred overnight at room temperature. At this point the reaction was concentrated *in vacuo* then taken up in diethyl ether and stirred/sonicated alternately until solids formed. The mixture was stirred vigorously at room temperature then filtered to isolate the solid product, which was used without further purification. To this material (100 mg, 129.9 μmol) was added HATU (74.1 mg, 1.5 equiv, 194.8 μmol), DMF (2 mL), (S,S,S)-AHPC hydrochloride (78.9 mg, 1.3 equiv, 168.8 μmol), and DIPEA (113 μL, 5 equiv, 649.3 μmol). The solution was stirred overnight at room temperature, at which point it was directly purified by reverse phase preparatory HPLC (10-90% MeCN in water w/0.1% TFA) to afford PROTAC **6286** as a fluffy white solid (46 mg, 25%). ^1H NMR (500 MHz, DMSO) δ 8.92 (s, 2H), 8.55 (t, *J* = 6.1 Hz, 1H), 8.18 (br sf, 1H), 7.77 (d, *J* = 8.9 Hz, 1H), 7.71 (d, *J* = 7.7 Hz, 2H), 7.50 (d, *J* = 8.0 Hz, 2H), 7.38 – 7.30 (m, 4H), 5.81 (d, *J* = 1.0 Hz, 1H), 4.39 (dd, *J* = 7.8, 3.0 Hz, 2H), 4.37 – 4.27 (m, 4H), 4.22 (dd, *J* = 14.1, 5.4 Hz, 3H), 4.19 – 4.11 (m, 1H), 4.00 (br s, 1H), 3.87 (dd, *J* = 10.1, 5.7 Hz, 1H), 3.78 (d, *J* = 11.2 Hz, 2H), 3.37 (dd, *J* = 10.0, 5.4 Hz, 1H), 3.29 (br s, 3H), 3.19 (td, *J* = 11.6, 2.3 Hz, 3H), 3.00 (br s, 1H), 2.86 (br s, 2H), 2.43 (p, *J* = 1.9 Hz, 6H), 2.37 (s, 3H), 2.30 – 2.24 (m, 3H), 2.21 (s, 3H), 2.15 (s, 3H), 2.07 – 2.02 (m, 4H), 1.68 (dt, *J* = 12.4, 6.0 Hz, 1H), 1.61 (br s, 1H), 1.52 (br s, 2H), 1.47 – 1.32 (m, 4H), 1.22 – 1.11 (m, 10H), 0.88 (s, 9H), 0.78 (t, *J* = 6.9 Hz, 3H). ^{13}C NMR (126 MHz, DMSO) δ 172.43 (d, *J* = 2.5 Hz), 170.94, 169.97, 162.99, 158.28 (q, *J* = 35.9 Hz), 151.51, 149.64, 147.70, 142.87, 139.23, 131.89, 131.15, 130.04, 129.71, 129.46, 128.66, 127.44, 127.09, 121.52, 114.64 (q, *J* = 291.6 Hz), 107.44, 69.10, 66.19, 58.50, 56.59, 55.57, 50.83, 50.39, 41.78, 37.90, 36.95, 34.91, 34.75, 34.67, 31.89, 28.86 (d, *J* = 6.0 Hz), 28.74 (d, *J* = 2.7 Hz), 28.64, 26.38, 25.40, 24.52, 18.97, 18.19, 15.92, 14.64. LRMS (ESI+): m/z calcd for $C_{67}H_{93}N_9O_8S$: 592 $[M+2H]^{+2}$; found: 592 $[M+2H]^{+2}$.

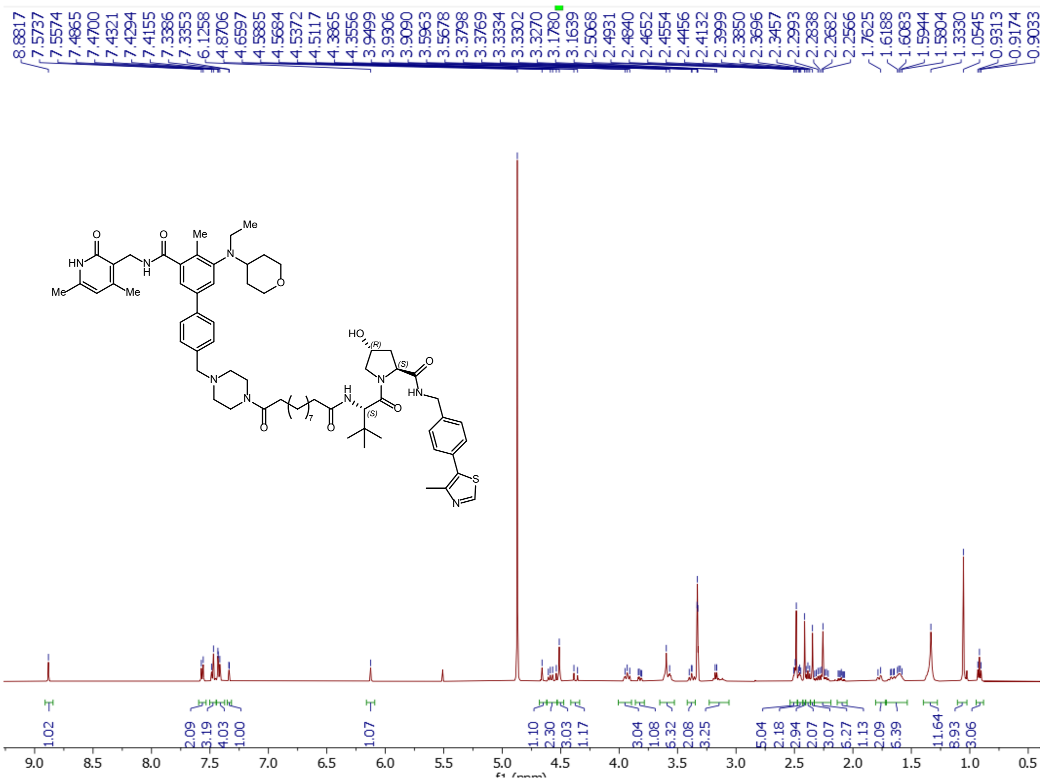

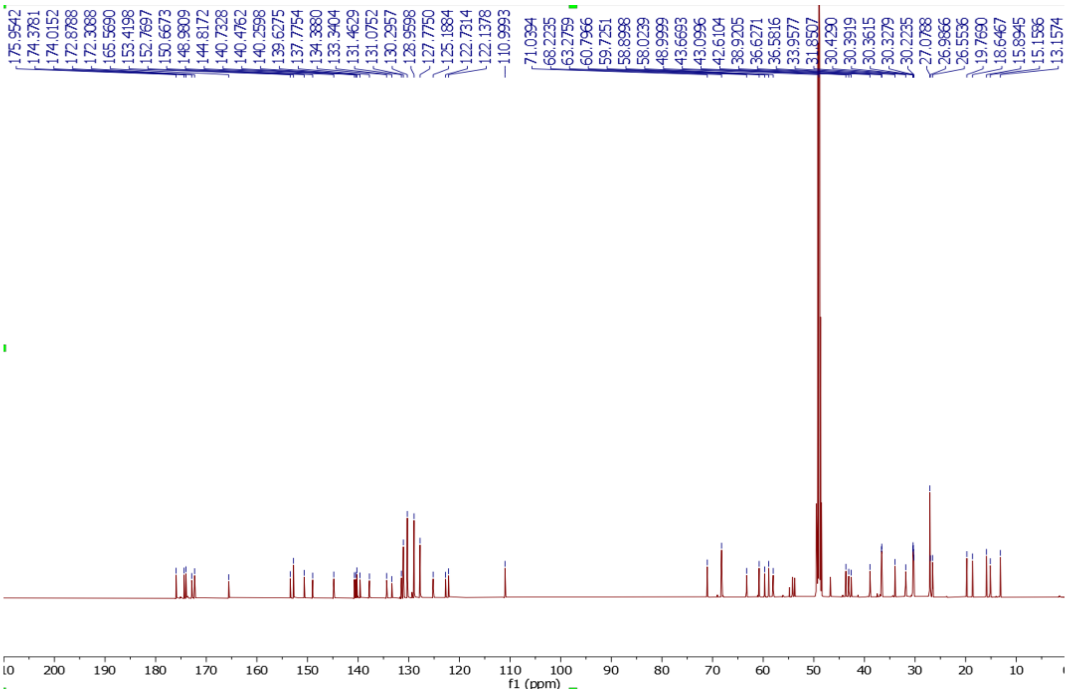

^{1}H- and ^{13}C-NMR spectra of PROTAC 6272

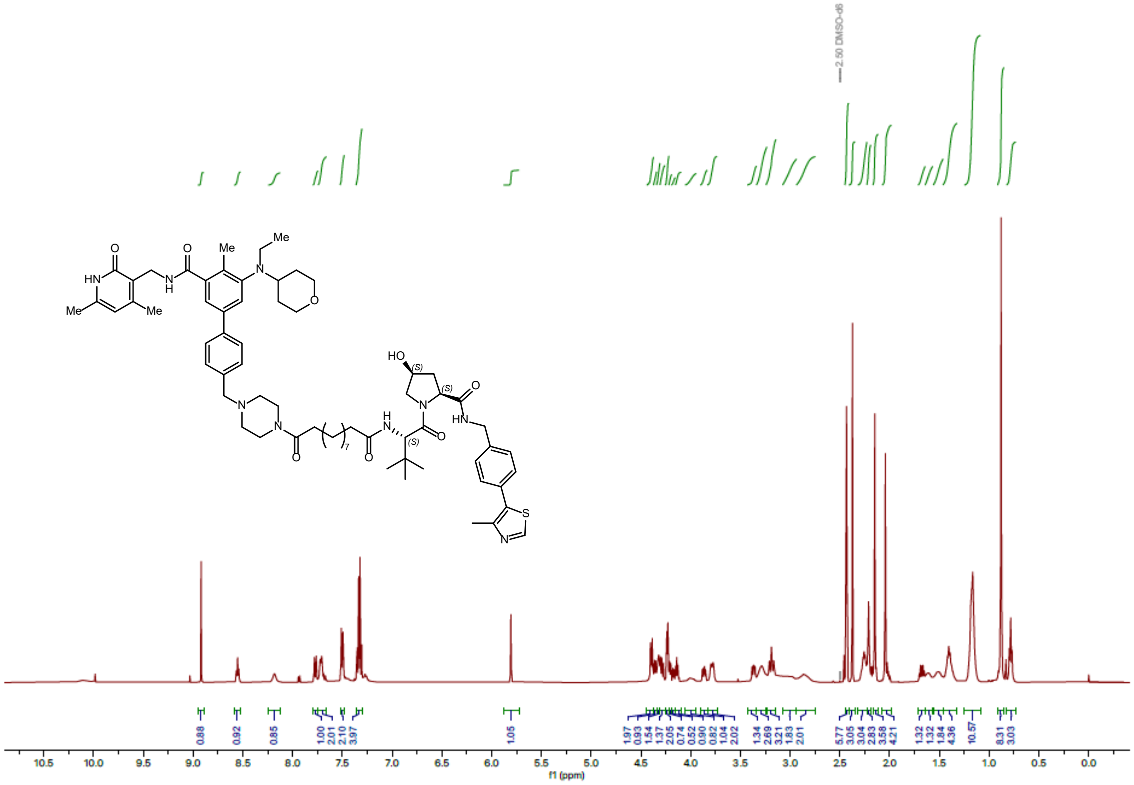

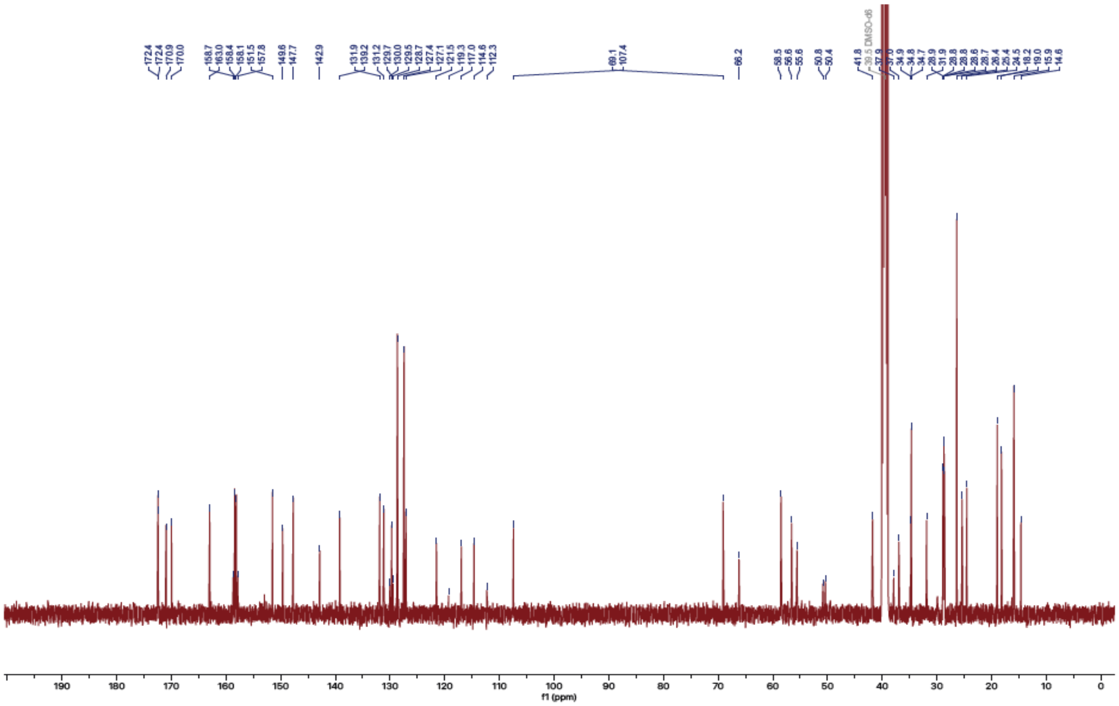

^{1}H- and ^{13}C-NMR spectra of PROTAC 6286.

Method S2: diaPASEF quantitative LCMS proteomics and data analysis

C4-2B or 22Rv1 cells (about 5 million cells per treatment), in triplicate, were treated with DMSO or 100 nM of PROTAC-6272 for 6 hrs. Cells were lysed by addition of lysis buffer (8 M Urea, 50 mM NaCl, 50 mM 4-(2-hydroxyethyl)-1-piperazineethanesulfonic acid (EPPS) pH 8.5, Protease and Phosphatase inhibitors) and homogenization by bead beating (BioSpec) for three repeats of 30 seconds at 2400 strokes/min. Bradford assay was used to determine the final protein concentration in the clarified cell lysate. Fifty micrograms of protein for each sample was reduced, alkylated, and precipitated using methanol/chloroform as previously described[3] and the resulting washed precipitated protein was allowed to air dry. Precipitated protein was resuspended in 4 M urea, 50 mM HEPES pH 7.4, followed by dilution to 1 M urea with the addition of 200 mM EPPS, pH 8. Proteins were digested with the addition of LysC (1:50; enzyme:protein) and trypsin (1:50; enzyme:protein) for 12 h at 37 °C. Sample digests were acidified with formic acid to a pH of 2-3 before desalting using C18 solid phase extraction plates (SOLA, Thermo Fisher Scientific). Desalted peptides were dried in a vacuum-centrifuge and reconstituted in 0.1% formic acid for liquid chromatography-mass spectrometry analysis.

LC-MS data were collected using a TimsTOF Pro2 (Bruker Daltonics, Bremen, Germany) coupled to a nanoElute LC pump (Bruker Daltonics, Bremen, Germany) via a CaptiveSpray nano-electrospray source. Peptides were separated on a reversed-phase C_{18} column (25 cm x 75 µm ID, 1.6 µM, IonOpticks, Australia) containing an integrated captive spray emitter. Peptides were separated using a 50 min gradient of 2 - 30% buffer B (acetonitrile in 0.1% formic acid) with a flow rate of 250 nL/min and column temperature maintained at 50 ºC. Data were collected using a diaPASEF acquisition method where the precursor distribution in the DDA *m/z*-ion mobility plane was used to design an acquisition scheme for Data-independent acquisition (DIA) data collection which included two windows in each 50 ms diaPASEF scan. Data was acquired using sixteen of these 25 Da precursor double window scans (creating 32 windows) which covered the diagonal scan line for doubly and triply charged precursors, with singly charged precursors able to be excluded by their position in the m/z-ion mobility plane. These precursor isolation windows were defined between 400 - 1200 *m/z* and 1/k0 of 0.7 - 1.3 V.s/cm^2.

The diaPASEF raw file processing and controlling peptide and protein level false discovery rates, assembling proteins from peptides, and protein quantification from peptides were performed using a cell line specific spectral library, or library free analysis in DIA-NN 1.8 searching against a Swissprot human database [4] (January 2021). Database search criteria largely followed the default settings for directDIA including tryptic with two missed cleavages, carbamidomethylation of cysteine, and oxidation of methionine and precursor Q-value (FDR) cut-off of 0.01. Precursor quantification strategy was set to Robust LC (high accuracy) with RT-dependent cross run normalization. Proteins with low sum of abundance (<2,000 x no. of treatments) were excluded from further analysis and resulting data was filtered to only include proteins that had a minimum

of 3 counts in at least 4 replicates of each independent comparison of treatment sample to the DMSO control. Proteins with missing values were imputed by random selection from a Gaussian distribution either with a mean of the non-missing values for that treatment group or with a mean equal to the median of the background (in cases when all values for a treatment group are missing) using in-house scripts in the R framework (R Development Core Team, 2014) [5,6]. Significant changes comparing the relative protein abundance of these treatments to DMSO control comparisons were assessed by two-sided moderated t-test as implemented in the limma package within the R framework[7].

Supplementary Figures:

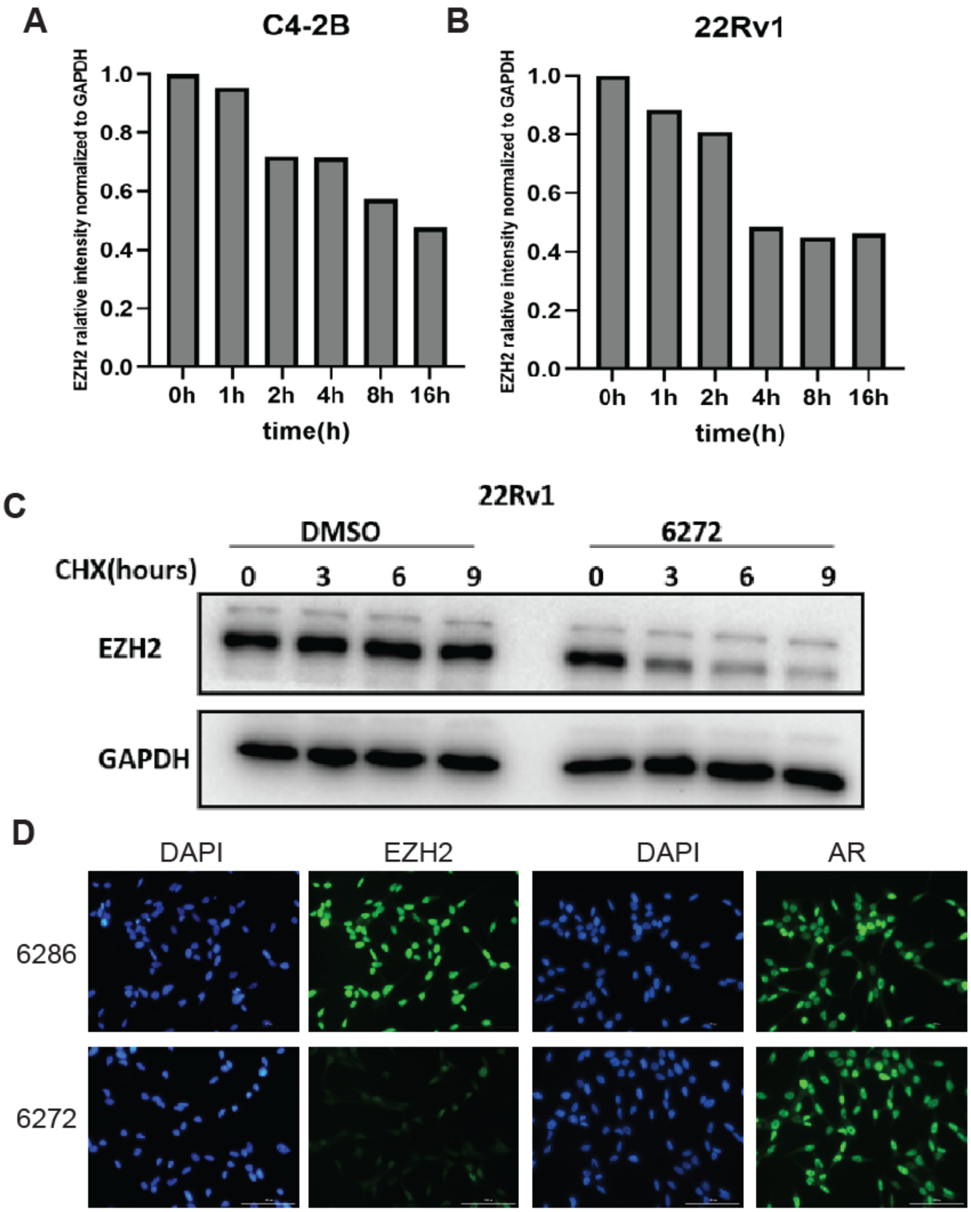

Fig. S1. EZH2 degradation by PROTAC-6272

A-B. Quantification of WB that is shown in Figure 1G.

C. Cycloheximide (CHX) chase assay to examine EZH2 half-life. 22Rv1 cells were treated with DMSO or PROTAC-6272 and Cycloheximide to examine EZH2 half-life. Cells were then harvested at different time points (0, 3, 6, and 9 hours) and processed for immunoblotting.

D. C4-2B cells were treated with 1 μM of negative control PROTAC-6286 or PROTAC-6272 for 72 hours, followed by immunofluorescence analysis.

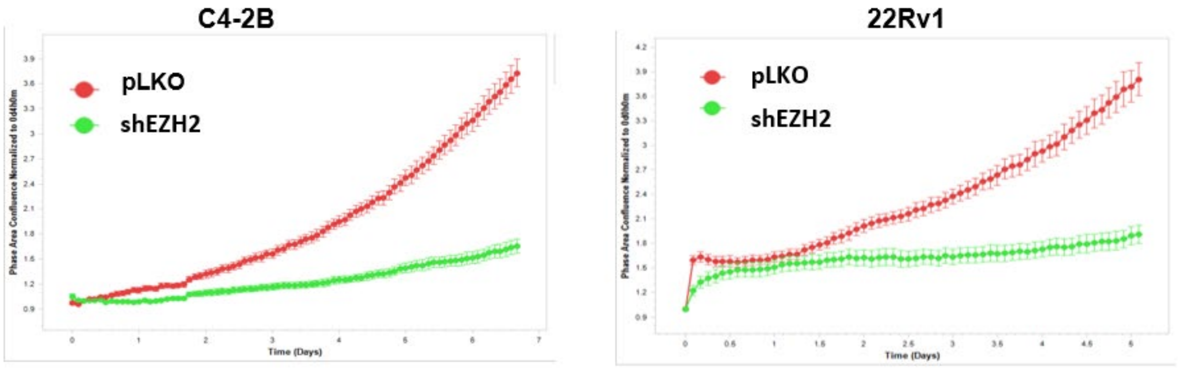

Fig. S2. Cell proliferation assays to test EZH2 knockdown in C4-2B and 22Rv1 cells
C4-2B and 22Rv1 cells were infected with control shRNA (pLKO) or shEZH2, then incubated in an IncuCyte live imager chamber; images were taken every 2 hours for up to 5 days.

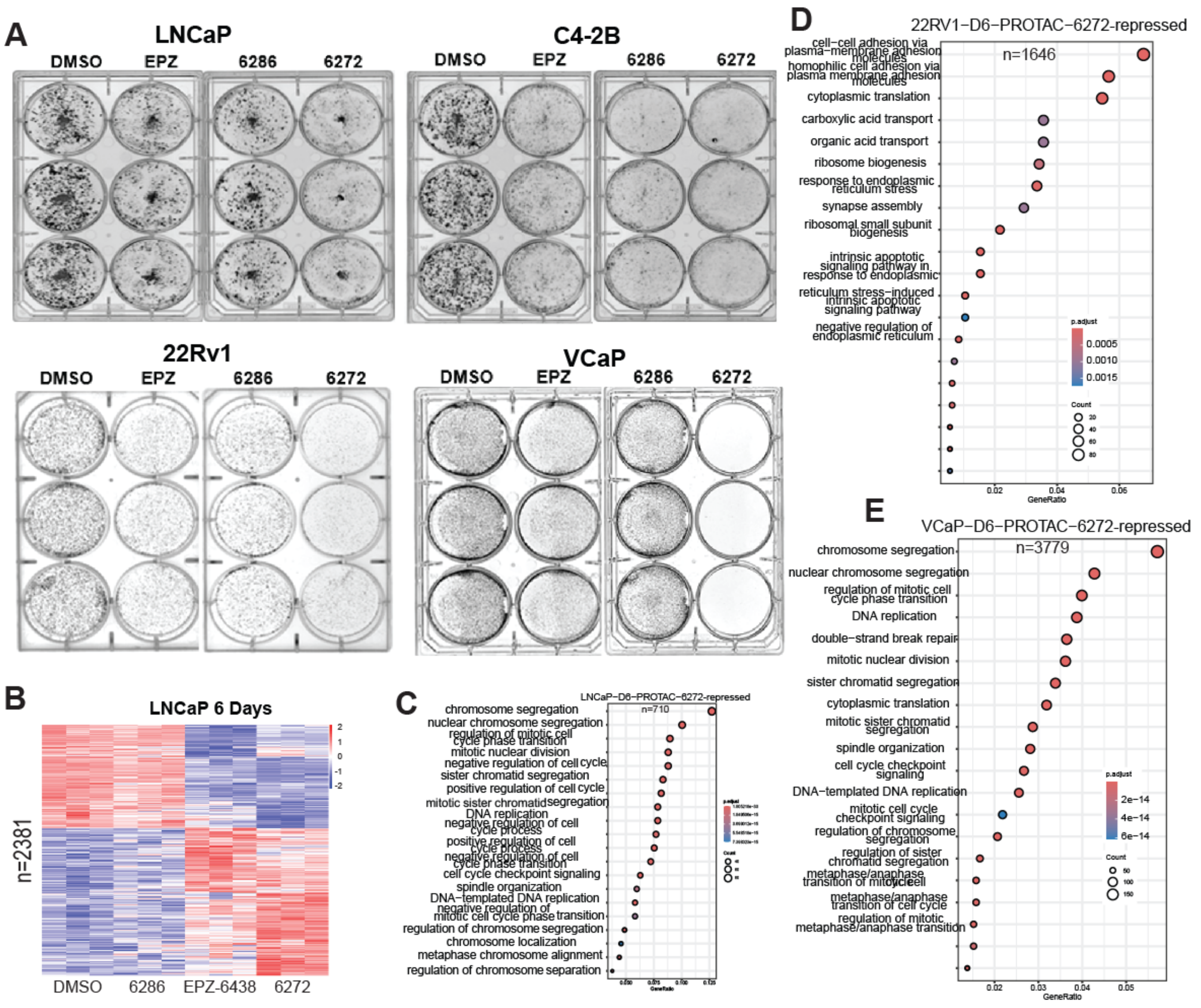

Fig. S3. Colony formation and RNA-seq analysis of various PCa cell lines

A. PCa cells were treated with DMSO, EPZ-6438, 6286 or PROTAC-6272(1 μM) for 2 weeks, followed by 0.002% crystal violet staining to assay colony formation. Data shown are technical replicates from one representative experiment.

B. LNCaP cells were subjected to triplicate RNA-seq analysis upon 6 days of treatment by DMSO, 6286, PROTAC-6272, and EPZ-6438(1 μM). Heatmap shows combined differentially expressed genes identified (with $|Log2FC| \geq 0.585$ and adjusted $p < 0.05$) by treatment of 6286, EPZ-6438, or PROTAC-6272 relative to DMSO.

C. Significantly downregulated genes were identified (n = 710, with padj < 0.05 and log2FC < –0.585) in LNCaP cells after 6 days of PROTAC-6272 (1 μM) treatment and subjected to Gene Ontology analyses.

D. Significantly downregulated genes were identified (n = 1,646, with padj < 0.05 and log2FC < –0.585,) in 22Rv1 cells after 6 days of PROTAC-6272 (1 μM) treatment and subjected to Gene Ontology analyses.

E. Significantly downregulated genes were identified (n = 3,779, with padj < 0.05 and log2FC < – 0.585,) in VCaP cells after 6 days of PROTAC-6272 (1 μM) treatment and subjected to Gene Ontology analyses.

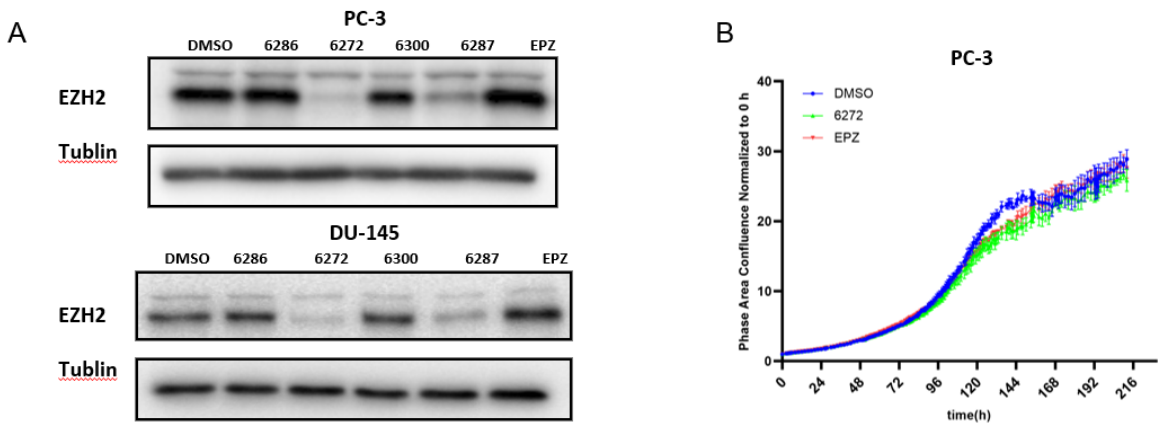

Fig. S4. Effect of PROTAC-6272 in AR-negative cells

A. PC-3 and DU145 cells were treated with 0.5μM of PROTAC-6272 and PROTAC-6287, and their respective negative controls, 6286 and 6300, for 6 days. Protein lysates were collected and then subjected to Immunoblot analysis.

B. PC-3 cells were treated with DMSO, PROTAC-6272 (1 μM), and EPZ-6438 (1 μM), then incubated in an IncuCyte live imager chamber, and images were taken every 2 hours up to 9 days. Cell proliferation rates were analyzed by IncuCyte live imager software.

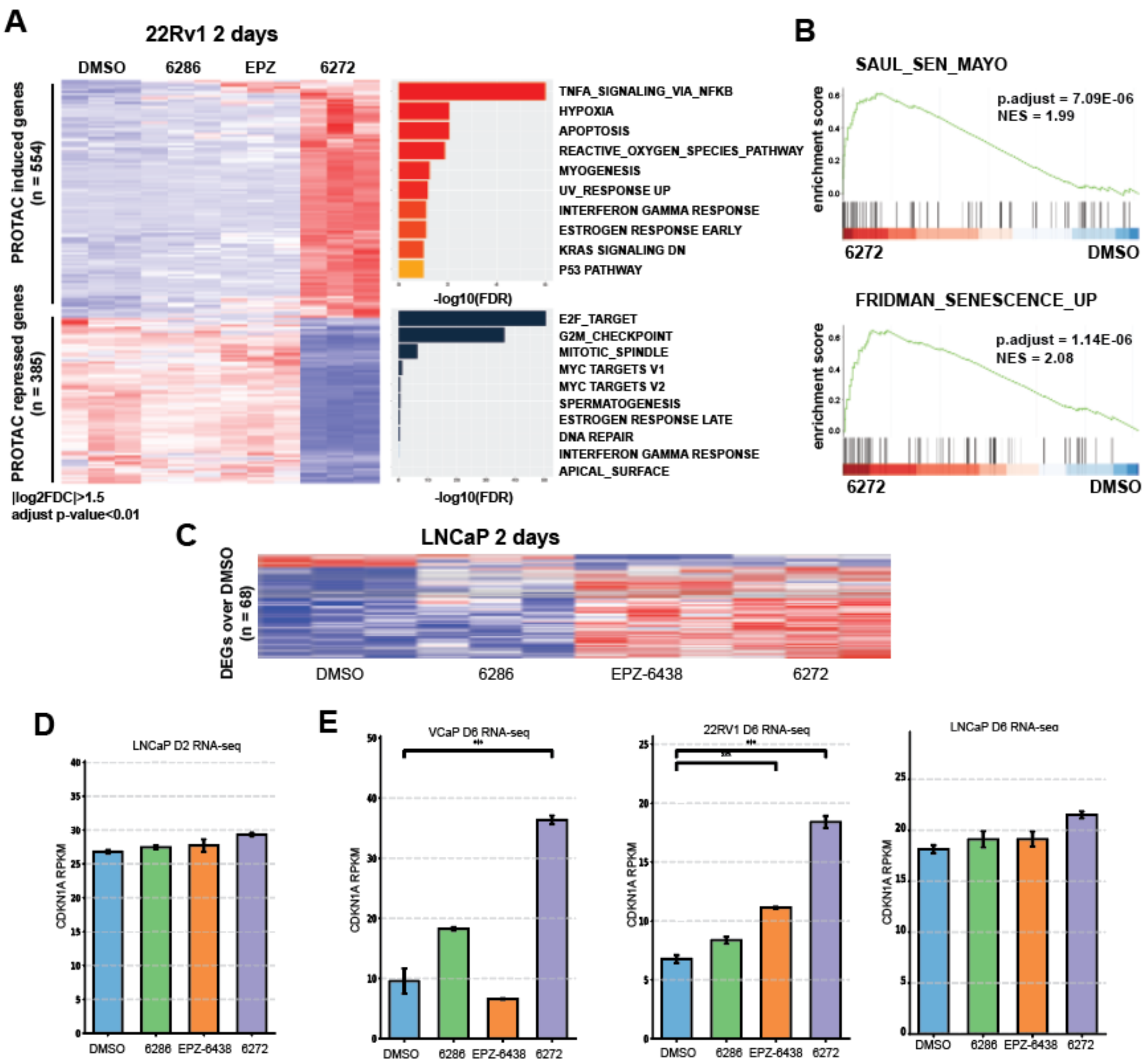

Fig. S5. RNA-seq data of PCa cells

A. 22Rv1 cells were subjected to triplicate RNA-seq analysis upon 48 hours treatment with DMSO, 6286, 6272 and EPZ-6438 (1uM). Heatmap shows differential express gene regulated by PROTAC-6272.Gene ontology (GO) analysis reveals top 10 HALLMARK concepts induced and repressed by PROTAC-6272.

B. Gene Set Enrichment Analysis (GSEA) was performed to determine the enrichment of two public cellular senescence gene sets, SAUL_SEN_MAYO (n=124) and FRIDMAN_SENESCENCE_UP (n=77), in gene expression dataset profiling identified in (A).

C. LNCaP cells were subjected to triplicate RNA-seq analysis upon 2 days of treatment with DMSO, 6286, PROTAC-6272, or EPZ-6438 (1 µM). Heatmap shows combined differentially expressed genes identified (with |Log2FC|≥0.585 and adjusted *p*<0.05) from treatment of 6286, EPZ-6438, or PROTAC-6272 relative to DMSO.

D. LNCaP cells were treated with DMSO, 6286, PROTAC-6272, or EPZ-6438 (1 µM) for 2 days. CDKN1A (p21) expression was quantified from RNA-seq data using RPKM (***p < 0.001).

E. VCaP, 22Rv1, and LNCaP cells were treated with DMSO, 6286, PROTAC-6272, or EPZ-6438 (1 μM) for 6 days. CDKN1A (p21) expression was quantified from RNA-seq data using RPKM (***p < 0.001).

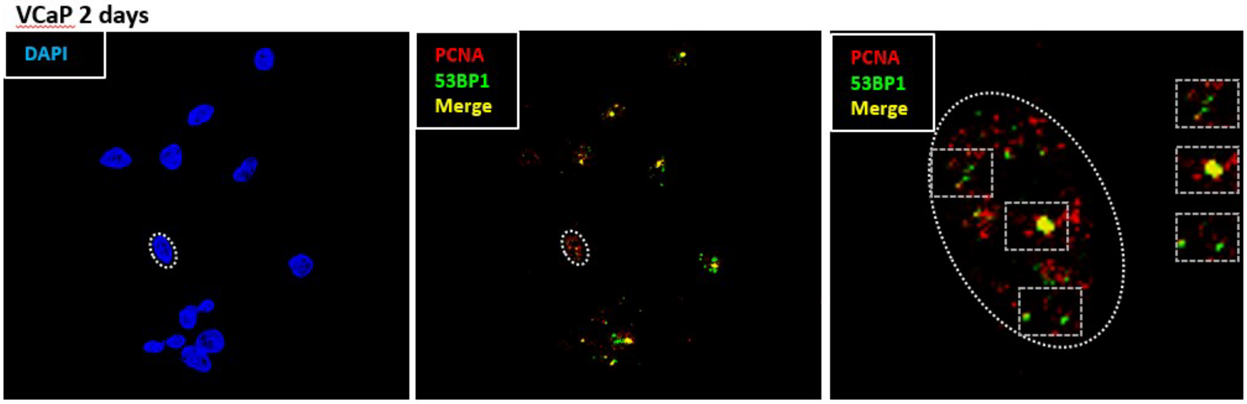

Fig. S6. PCNA and 53BP1 IF staining in VCaP cells

VCaP cells were treated with PROTAC-6272 and stained with PCNA (red) and 53BP1 (green). DAPI staining is shown in blue, and merged PCNA and 53BP staining are shown in yellow signals. Right: magnified views of the indicated cell.

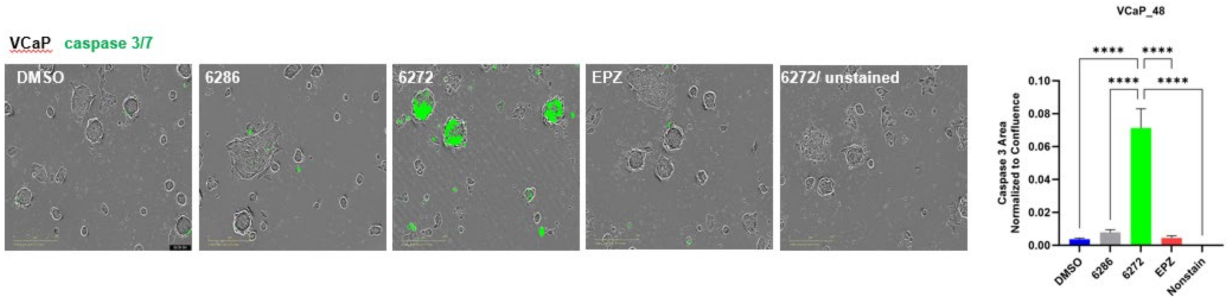

Fig. S7. Caspase3/7 fluorescence staining to test apoptosis in VCaP cells

Caspase3/7 fluorescence staining was performed in VCaP cells with DMSO,6286, PROTAC-6272, and EPZ-6438 (1 μM) for 48 hours, and the apoptosis signal was captured and analyzed by IncuCyte live imager.

Reference :

1 Liu, Z. *et al.* Design and Synthesis of EZH2-Based PROTACs to Degrade the PRC2 Complex for Targeting the Noncatalytic Activity of EZH2. *J Med Chem* **64**, 2829–2848 (2021). https://doi.org/10.1021/acs.jmedchem.0c02234

2 Liu, Z. *et al.* Design and Synthesis of EZH2-Based PROTACs to Degrade the PRC2 Complex for Targeting the Noncatalytic Activity of EZH2. *Journal of Medicinal Chemistry* **64**, 2829–2848 (2021). https://doi.org/10.1021/acs.jmedchem.0c02234

3 Donovan, K. A. *et al.* Thalidomide promotes degradation of SALL4, a transcription factor implicated in Duane Radial Ray syndrome. *eLife* **7** (2018). https://doi.org/10.7554/eLife.38430

4 Demichev, V. *et al.* dia-PASEF data analysis using FragPipe and DIA-NN for deep proteomics of low sample amounts. *Nat Commun* **13**, 3944 (2022). https://doi.org/10.1038/s41467-022-31492-0

5 Baek, K. *et al.* Unveiling the hidden interactome of CRBN molecular glues with chemoproteomics. *bioRxiv* (2024). https://doi.org/10.1101/2024.09.11.612438

6 Demichev, V., Messner, C. B., Vernardis, S. I., Lilley, K. S. & Ralser, M. DIA-NN: neural networks and interference correction enable deep proteome coverage in high throughput. *Nat Methods* **17**, 41–44 (2020). https://doi.org/10.1038/s41592-019-0638-x

7 Ritchie, M. E. *et al.* limma powers differential expression analyses for RNA-sequencing and microarray studies. *Nucleic Acids Res* **43**, e47 (2015). https://doi.org/10.1093/nar/gkv007
